# Supplementary material for: Loss of kallikrein‐related peptidase 7 exacerbates amyloid pathology in Alzheimer's disease model mice
Source: EMBO Mol Med. 2018 Jan 8;10(3):e8184. doi: 10.15252/emmm.201708184 (PMC5840542; doi:10.15252/emmm.201708184)
Supplement: Supplementary file 1 — Appendix [file EMMM-10-e8184-s001.pdf]

Appendix

Supplemental Materials for

Loss of kallikrein-related peptidase 7 exacerbates amyloid pathology in Alzheimer's disease model mice

Kiwami Kidana, Takuya Tatebe, Kaori Ito, Norikazu Hara, Akiyoshi Kakita, Takashi Saito, Sho Takatori, Yasuyoshi Ouchi, Takeshi Ikeuchi, Mitsuhiro Makino, Takaomi C. Saido, Masahiro Akishita, Takeshi Iwatsubo, Yukiko Hori and Taisuke Tomita

Table of contents

	Page
Appendix Fig. S1	2
Appendix Fig. S2	4
Appendix Fig. S3	6
Appendix Fig. S4	8
Appendix Fig. S5	9
Appendix Fig. S6	11
Appendix Fig. S7	12
Appendix Fig. S8	14
Appendix Fig. S9	16
Appendix Fig. S10	17
Appendix Table S1	18

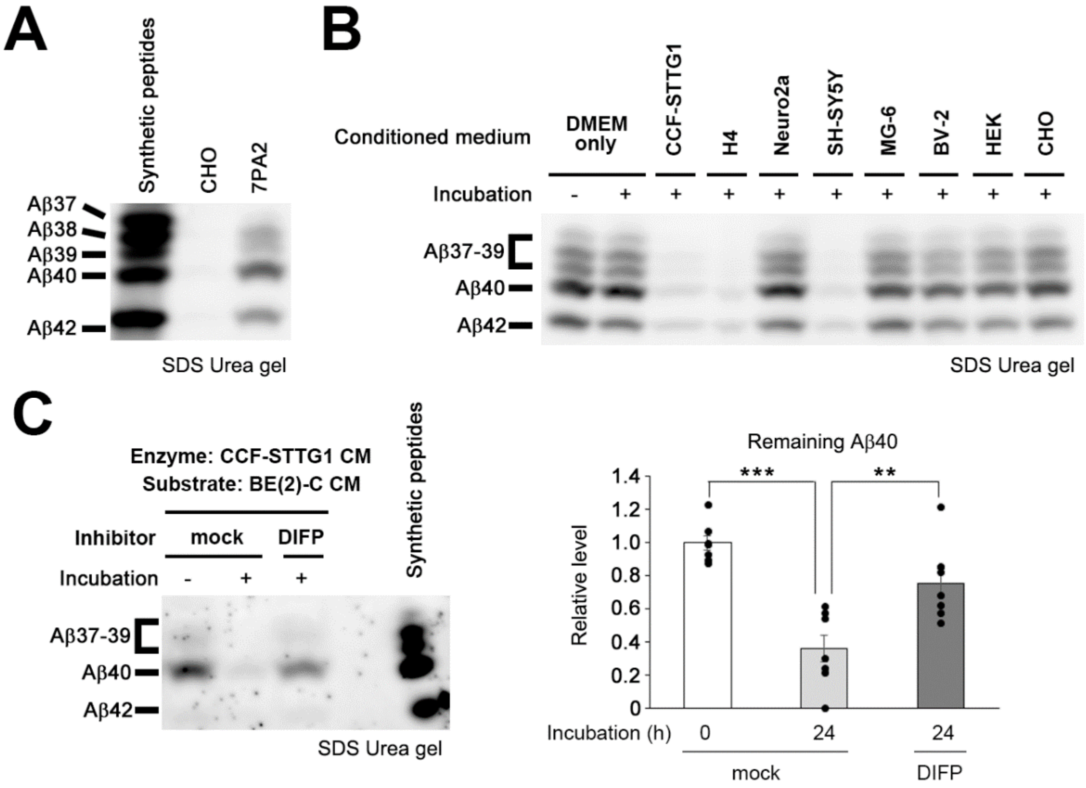

Appendix Fig. S1. Aβ degradation activity in the conditioned medium from cultured cells

(A) Biochemical characterization of the Aβ species secreted from 7PA2 cells using urea-SDS PAGE analysis. A mixture of synthetic Aβ peptides (i.e., Aβ37, Aβ38, Aβ39, Aβ40 and Aβ42) was loaded on the left lane. In this SDS-PAGE system, longer Aβ species migrates faster than short Aβ (B) The conditioned media from various cell lines was incubated for 24 hours with the conditioned medium of 7PA2 cells. Remaining Aβ the mixture was visualized by immunoblotting. (C) Aβ degrading activity of the conditioned medium of CCF-STTG1 cells against naïve human Aβ species secreted from the human neuroblastoma cell line, BE(2)-C cells. Quantification of relative remaining Aβ40 in the

mixture of normal culture medium and conditioned medium is shown left the blot ($n = 7$, mean ± s.e.m. (**) for $p < 0.01$, and (***) for $p < 0.001$ by Tukey's test).

A

NP sample set	*Ctrl*	*AD*	P-*value*
N	24	29	-
AAD, yr	71.6 ± 6.8	79.6 ± 8.6	0.001 [1]
Gender, (F : M)	10 : 14	19 : 10	0.102 [2]
PMI, hr	4.3 ± 4.8	3.8 ± 1.7	0.124 [1]
BW, g	1024 ± 189	1107 ± 167	0.127 [1]
APOE			
Genotype, (ε2*3 : ε3*3 : ε3*4 : ε4*4)	1 : 20 : 3 : 0	3 : 14 : 9 : 3	0.031 [3]
Allele, (ε2 : ε3 : ε4)	1 : 44 : 3	3 : 40 : 15	0.009 [3]

[1] Calculated by Mann-Whitney *U*-test between AD and Ctrl;
[2] Calculated by Fisher's exact test for gender distribution;
[3] Calculated by Fisher's exact test for *APOE* ε4 allele carrier status (ε4 carrier and ε4 non-carrier).

B

Gene	Detector	Status	Mean	SD
GUSB	Hs99999908_m1	AD	28.413	0.518
GUSB	Hs99999908_m1	Ctrl	28.422	0.526
RPS17	Hs00734303_g1	AD	25.519	0.523
RPS17	Hs00734303_g1	Ctrl	25.298	0.459
CASC3	Hs00201226_m1	AD	25.464	0.527
CASC3	Hs00201226_m1	Ctrl	25.301	0.448
KLK7	Hs01012730_g1	AD	31.643	1.640
KLK7	Hs01012730_g1	Ctrl	30.115	1.260
KLK7	Hs00192503_m1	AD	30.149	1.672
KLK7	Hs00192503_m1	Ctrl	28.576	1.374
KLK7	Hs01012731_m1	AD	30.450	1.623
KLK7	Hs01012731_m1	Ctrl	29.017	1.253

C

Gene	Detector	Controls	AD	log2FC	*P*-value
KLK7	Hs01012730_g1	30.115	31.643	1.528	0.000086
KLK7	Hs00192503_m1	28.576	30.149	1.573	0.000155
KLK7	Hs01012731_m1	29.016	30.450	1.434	0.000198
CASC3	Hs00201226_m1	25.301	25.464	0.164	0.156650
GUSB	Hs99999908_m1	28.422	28.413	-0.009	0.472468
RPS17	Hs00734303_g1	25.298	25.519	0.222	0.072336

D

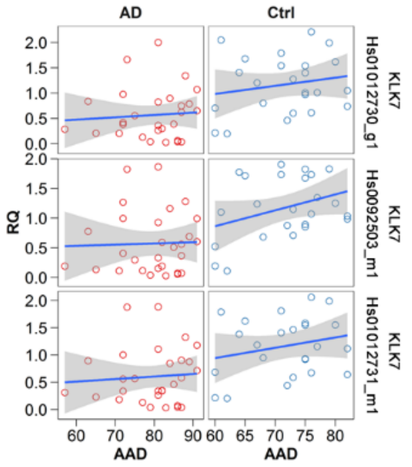

Detector	Gene	Status	cor_coeff	p_value
Hs01012730_g1	KLK7	AD	0.080	0.682
Hs01012730_g1	KLK7	Ctrl	0.193	0.366
Hs00192503_m1	KLK7	AD	0.033	0.867
Hs00192503_m1	KLK7	Ctrl	0.349	0.094
Hs01012731_m1	KLK7	AD	0.077	0.690
Hs01012731_m1	KLK7	Ctrl	0.238	0.263

E

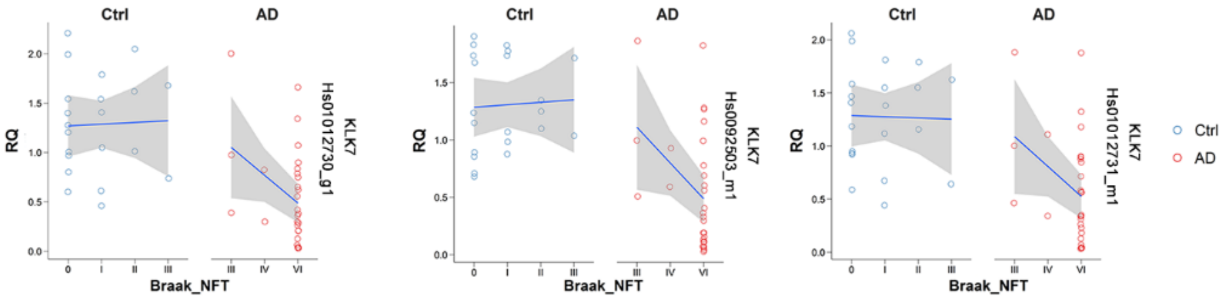

Gene	Detector	Status	cor_coeff	P_value
KLK7	Hs01012730_g1	AD	-0.351	0.062
KLK7	Hs01012730_g1	Ctrl	0.035	0.879
KLK7	Hs00192503_m1	AD	-0.379	0.042
KLK7	Hs00192503_m1	Ctrl	0.053	0.819
KLK7	Hs01012731_m1	AD	-0.346	0.066
KLK7	Hs01012731_m1	Ctrl	-0.024	0.919

Appendix Fig. S2. Expression analyses of mRNAs in patients of human AD brains

(A) Information of control and AD patients analyzed in this study. Abbreviations are; AAD, age at death; *APOE*, apolipoprotein E; BW, brain weight; Ctrl, control; g, grams; hr, hours; F, female; M, male; PMI, postmortem interval; RIN, RNA integrity number; FC, frontal cortex; yr, years. Data are presented as the mean ± s.d. (B) Real-time Ct values of three internal control genes (*GUSB*, *RPS17*, and *CASC3*) and *KLK7* mRNA determined by the quantitative RT-PCR analysis. (C) Statistical analysis of (B) by Mann-Whitney *U*-test between Ctrl and AD. The expression of *GUSB* showed the most comparable values between AD patients and control subjects among the internal control genes. From this result, we chose *GUSB* as an endogenous mRNA control for standardization. (D) Age-dependent changes of relative *KLK7* mRNA expression. Trend of increased expression of *KLK7* in control brains was observed, although it was not statistically significant in our cohort. Correlation coefficient was calculated by Spearman's correlation test. (E) Comparison of the expression levels of *KLK7* mRNA with Braak neurofibrillary tangle stages. Correlation coefficient was calculated by Spearman's correlation test.

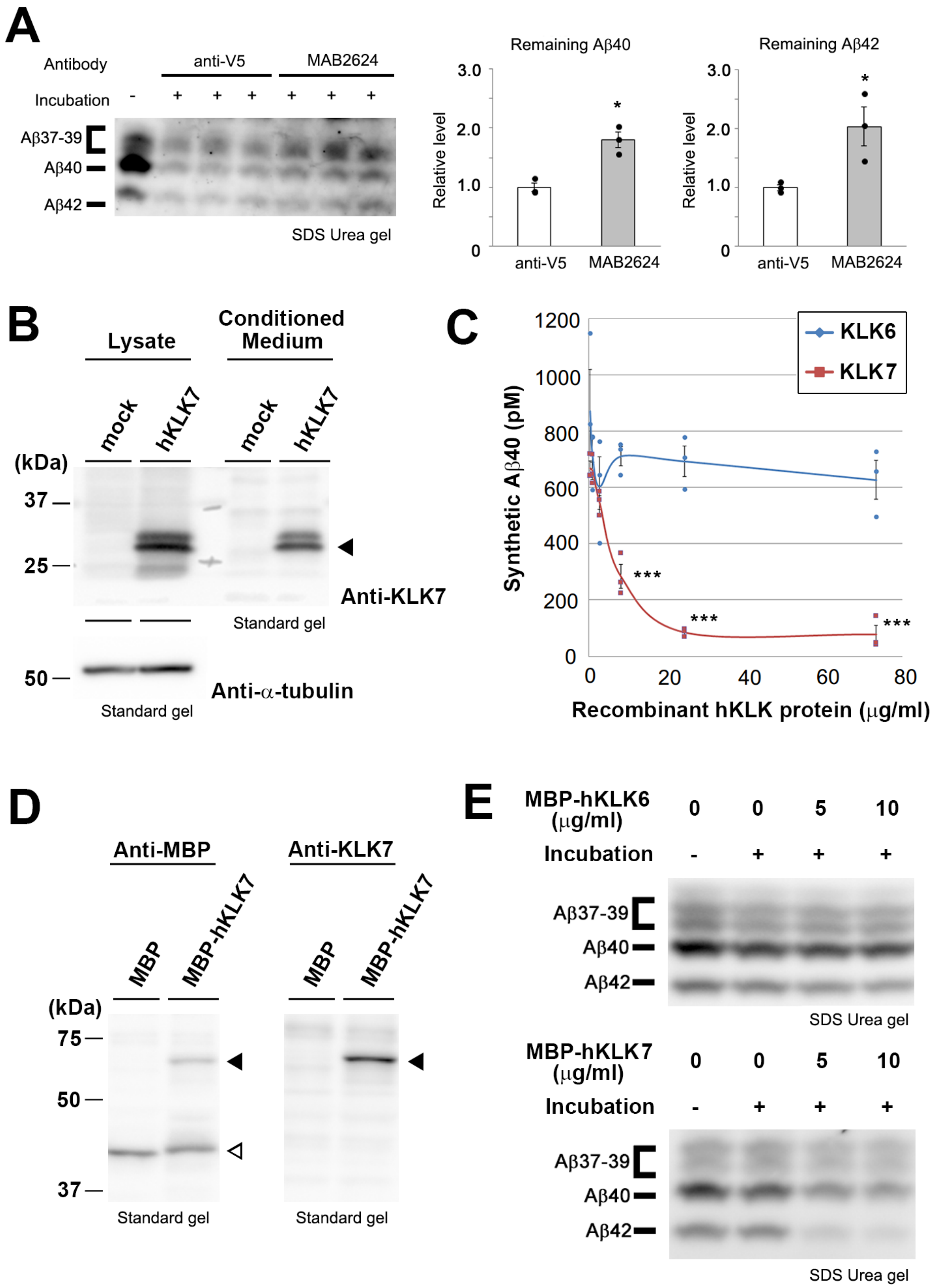
A
Antibody
anti-V5
MAB2624
Incubation
- + + + + + +
Aβ37-39
Aβ40
Aβ42
SDS Urea gel
Remaining Aβ40
Remaining Aβ42
Relative level
3.0
2.0
1.0
0
*
anti-V5
MAB2624
B
Lysate
Conditioned Medium
mock
hKLK7
(kDa)
37
25
50
Anti-KLK7
Standard gel
Anti-α-tubulin
C
KLK6
KLK7
Synthetic Aβ40 (pM)
1200
1000
800
600
400
200
0
20
40
60
80
***
Recombinant hKLK protein (μg/ml)
D
Anti-MBP
Anti-KLK7
MBP
MBP-hKLK7
75
E
MBP-hKLK6 (μg/ml)
0 0 5 10
Incubation
- + + +
MBP-hKLK7 (μg/ml)

Appendix Fig. S3. Characterization of the Aβ degradation activity of KLK7 protein

(A) Effect of the KLK7-neutralizing antibody MAB2624 and its isotype control (anti-V5 monoclonal antibody) on the Aβ degradation activity of CCF-STTG1. Quantification of relative remaining Aβ40 and Aβ42 in the mixture of normal culture medium and conditioned medium is shown left of the blot (n = 3, mean ± s.e.m. (*) for $p < 0.05$ by student *t*-test). (B) Immunoblot analysis of the conditioned medium of COS-1 cells expressing human KLK7 (hKLK7). (C) *In vitro* Aβ degradation by commercially available recombinant hKLK proteins expressed in mouse myeloma NS0 cell line (R&D systems). After 24 hours of incubation, the levels of synthetic Aβ40 peptides (625 pM before incubation) mixed with recombinant hKLK7 or KLK6 proteins were measured using sandwich ELISA (n = 3, mean ± s.e.m., (***) for $p < 0.001$ by Tukey's test). (D) Immunoblot analysis of the purified MBP-tagged hKLK7 protein (black arrowhead) expressed in *E. coli*. Purified MBP was used as a control protein (white arrowhead). Some MBP-hKLK7 proteins might be cleaved at the linker. (E) *In vitro* Aβ degradation by purified MBP-tagged hKLK proteins against Aβ secreted from the 7PA2 cells.

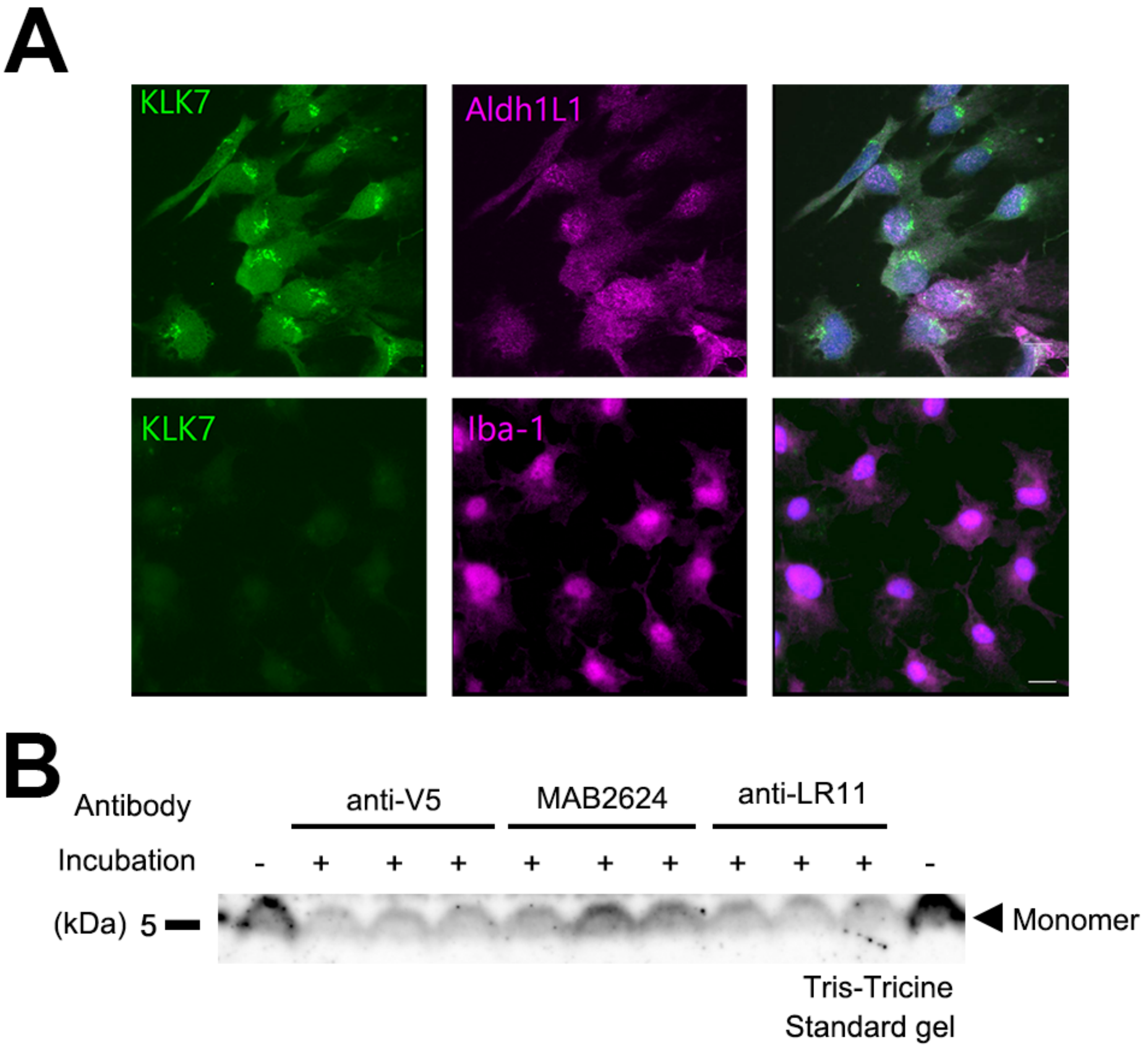

Appendix Fig. S4. Immunocytochemical analysis of Klk7 in primary astrocytes and microglia

(A) Primary astrocytes (upper panels) or microglia (lower panels) that were separately obtained from wild-type mice are shown (green, anti-KLK7 antibody; magenta, anti-Aldh1L1 or anti-Iba-1 antibody; blue, DAPI; scale bar, 10 μm). (B) Effect of the KLK7-neutralizing antibody MAB2624 and its isotype controls (anti-V5 and anti-LR11 monoclonal antibodies) on the Aβ degradation activity of primary astrocytes.

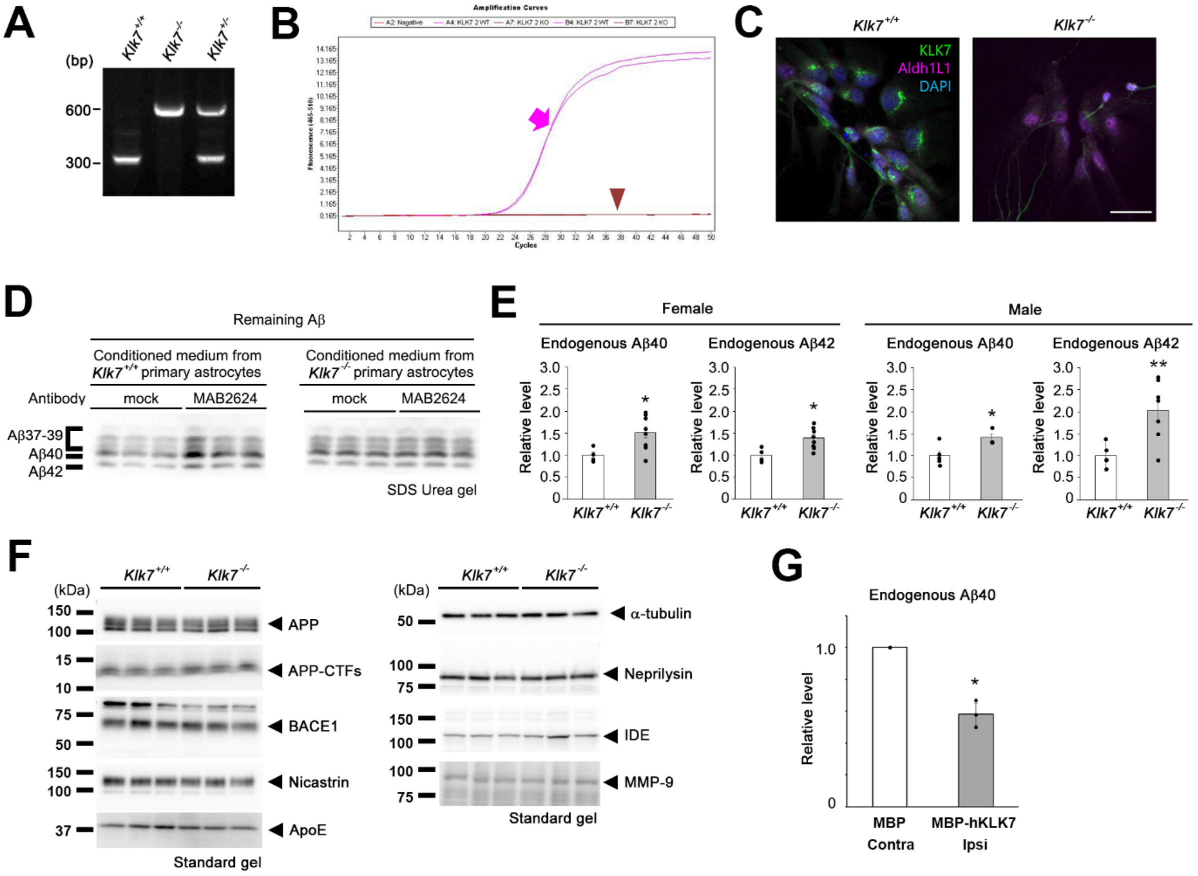

Appendix Fig. S5. Characterization of Klk7$^{-/-}$ mice and their brain Aβ levels

(A) Genotyping of male *Klk7*$^{+/+}$, *Klk7*$^{+/-}$ or *Klk7*$^{-/-}$ mice by PCR using genomic DNA obtained from tails. (B) Quantitative RT-PCR analysis of endogenous *Klk7* mRNA levels in the brains of *Klk7*$^{+/+}$ (pink, arrow) or *Klk7*$^{-/-}$ (brown, arrowhead) mice. (C) Immunocytochemical analysis of a primary neuron and astrocyte obtained from *Klk7*$^{+/+}$ or *Klk7*$^{-/-}$ mouse embryos (green, anti-KLK7 antibody; magenta, anti-Aldh1L1 antibody; blue, DAPI; scale bar, 50 µm). (D) Effect of the KLK7-neutralizing antibody MAB2624 on the Aβ degradation activity of primary astrocytes obtained from *Klk7*$^{+/+}$ or *Klk7*$^{-/-}$ mice. (E) Biochemical analyses of endogenous Tris buffer soluble murine Aβ in the brains of 3 months

of age female as well as male *Klk7*$^{+/+}$ or *Klk7*$^{-/-}$ mice. Relative levels of Aβ40 and Aβ42 were quantified by sandwich ELISA (n = 4 or 5, mean ± s.e.m., (*) for $p < 0.05$, (**) for $p < 0.01$ by student *t*-test). (F) Immunoblot analyses of APP, APP-CTFs, BACE1, nicastrin, ApoE, α-tubulin, neprilysin, insulin degrading enzyme (IDE) and matrix metalloprotease 9 (MMP-9) in the brains of 3 months of age male *Klk7*$^{+/+}$ or *Klk7*$^{-/-}$ mice. (G) Levels of endogenous Aβ40 in the hippocampi of wild-type mouse (5 months of age female) brains injected with MBP (contralateral side, contra) or MBP-KLK7 (ipsilateral side, ipsi). Note that 40% reduction of Aβ40 level was observed in the hippocampal region of ipsilateral side (n = 4, mean ± s.e.m., (**) for $p < 0.01$ by student *t*-test).

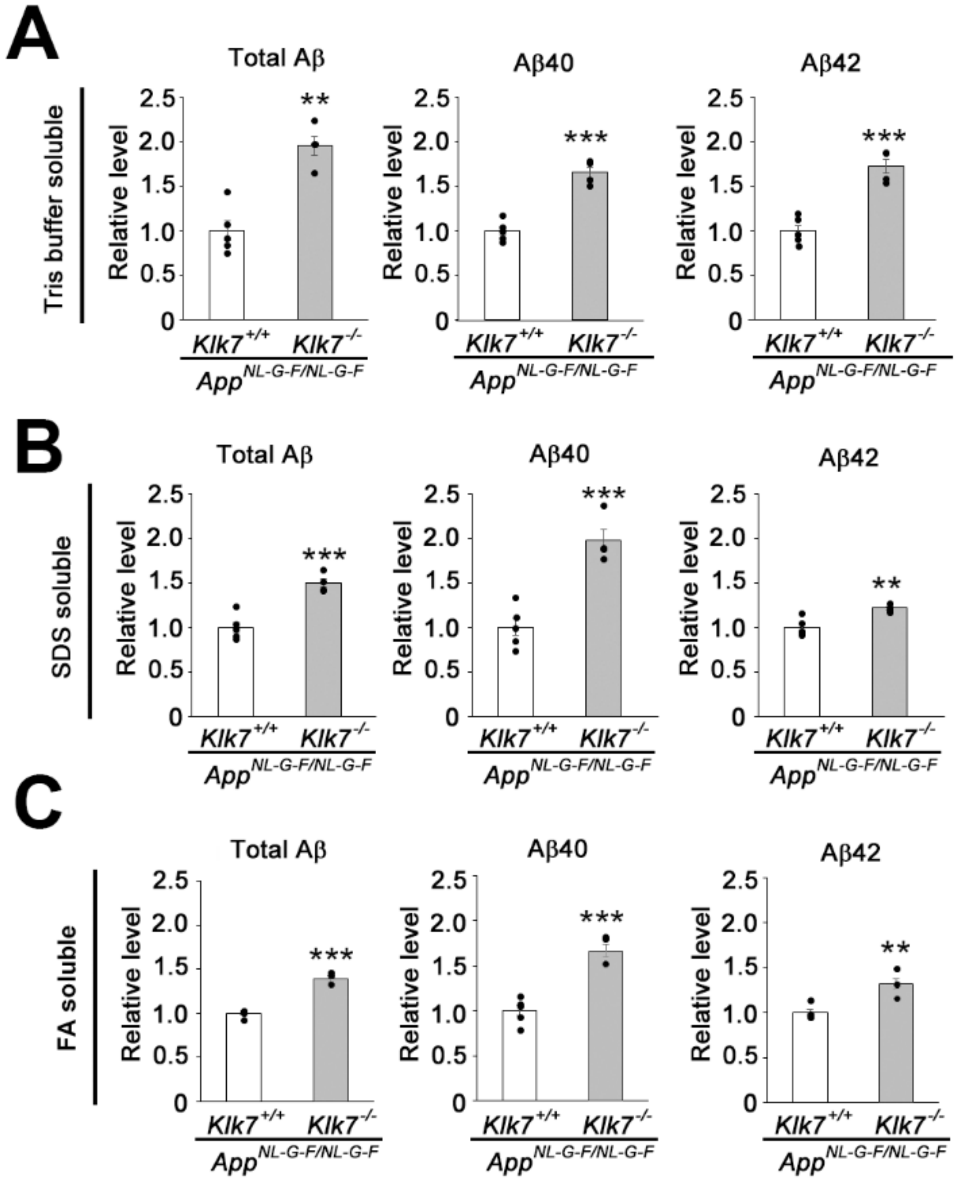

Appendix Fig. S6. Immunoblot analysis of the brains of App$^{NL\text{-}G\text{-}F/NL\text{-}G\text{-}F}$; Klk7$^{+/+}$ and App$^{NL\text{-}G\text{-}F/NL\text{-}G\text{-}F}$; Klk7$^{-/-}$ mice

(A-C) Quantification of the relative levels of Aβ in Tris buffer soluble (A), SDS soluble (B) and formic acid (FA) soluble (C) fractions in Figure 5. Relative levels of total Aβ, Aβ40 and Aβ42 species were quantified by immunoblot analyses (n = 4 or 5, mean ± s.e.m., (**) for $p < 0.01$, and (***) for $p < 0.001$ by student *t*-test).

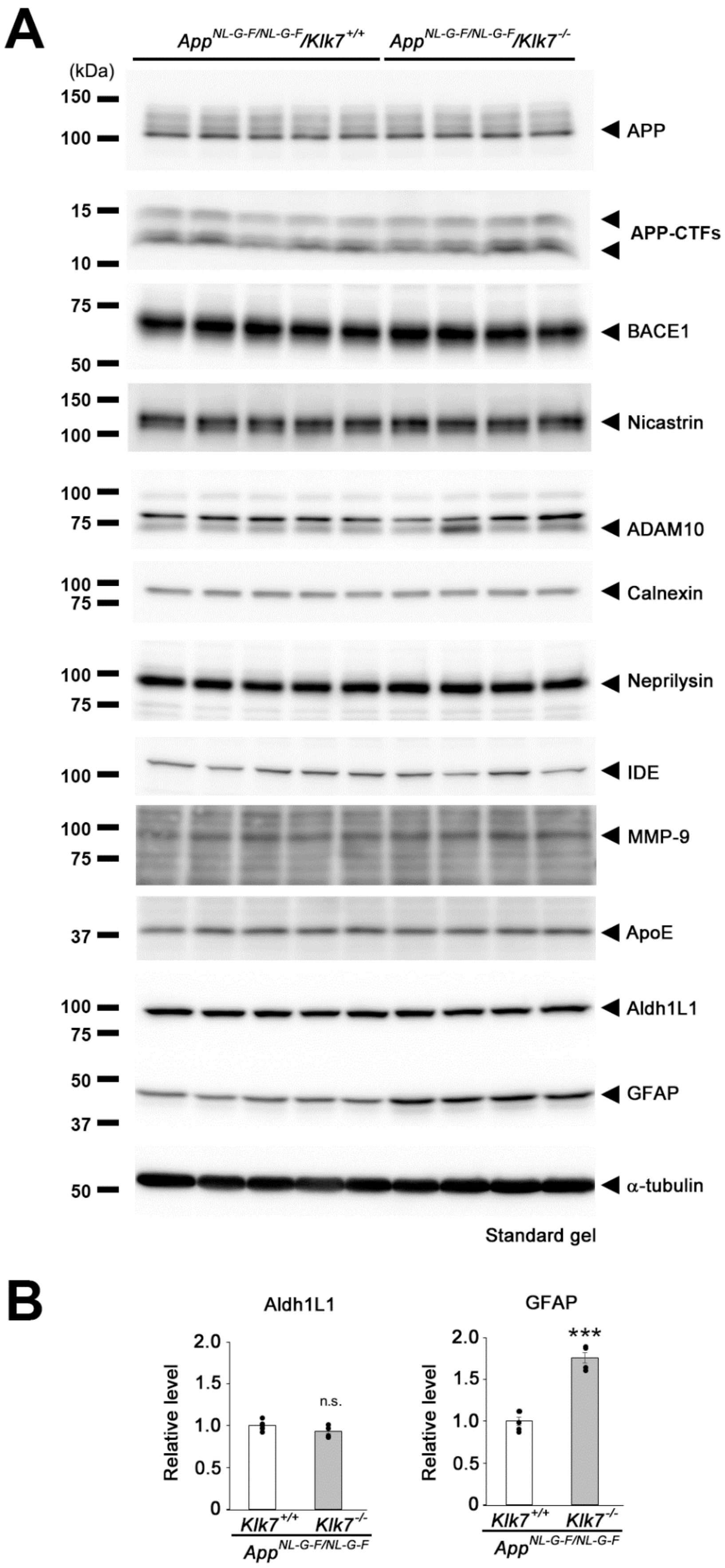
A
App^NL-G-F/NL-G-F/Klk7^+/+
App^NL-G-F/NL-G-F/Klk7^-/-
(kDa)
150
100
APP
15
10
APP-CTFs
75
50
BACE1
150
100
Nicastrin
100
75
ADAM10
100
75
Calnexin
100
75
Neprilysin
100
IDE
100
75
MMP-9
37
ApoE
100
75
Aldh1L1
50
37
GFAP
50
α-tubulin
Standard gel
B
Aldh1L1
GFAP
Relative level
2.0
1.5
1.0
0.5
0
n.s.
***
Klk7^+/+
Klk7^-/-
App^NL-G-F/NL-G-F

Appendix Fig. S7. Immunoblot analysis of the brains of male $App^{NL\text{-}G\text{-}F/NL\text{-}G\text{-}F}$; $Klk7^{+/+}$ and $App^{NL\text{-}G\text{-}F/NL\text{-}G\text{-}F}$; $Klk7^{-/-}$ mice

(A) Immunoblot analyses of APP, BACE1, nicastrin, ADAM10, calnexin, neprilysin, IDE, MMP-9, ApoE, Aldh1L1, GFAP and α-tubulin in the brains of 3 months of age male $App^{NL\text{-}G\text{-}F/NL\text{-}G\text{-}F}$; $Klk7^{+/+}$ and $App^{NL\text{-}G\text{-}F/NL\text{-}G\text{-}F}$; $Klk7^{-/-}$ mice. (B) Quantitative levels of Aldh1L1 and GFAP are shown ($n = 4$ or 5, mean ± s.e.m., (***) for $p < 0.001$ by student *t*-test).

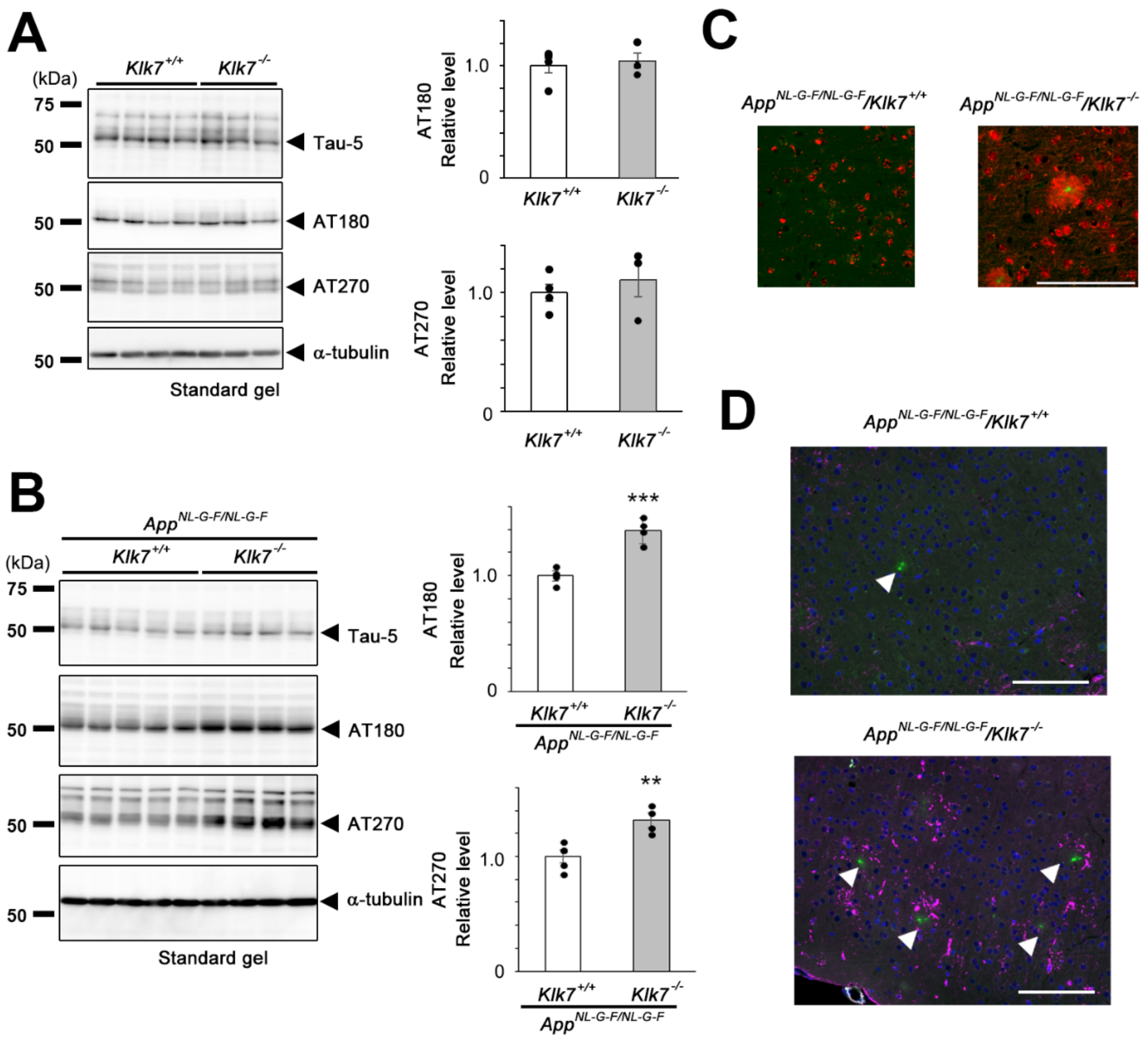

Appendix Fig. S8. Impact of Klk7 gene ablation on the phosphorylation of endogenous tau, astrogliosis and formation of dystrophic neurites

Phosphorylation of endogenous tau in the brains of 3-4 months of age male wild-type (*Klk7*$^{+/+}$), *Klk7*$^{-/-}$ (A) (n = 3 or 4, mean ± s.e.m.), *App*$^{NL\text{-}G\text{-}F/NL\text{-}G\text{-}F}$*; Klk7*$^{+/+}$ and *App*$^{NL\text{-}G\text{-}F/NL\text{-}G\text{-}F}$*; Klk7*$^{-/-}$ mice (B) (n = 4 or 5, mean ± s.e.m.) was examined by immunoblot analysis using Tris buffer soluble fractions. Relative levels of phospho-Thr231 (AT180) and phospho-Thr181 (AT270) normalized by total tau levels (Tau-5) are shown right of the blots. Note that

the endogenous tau phosphorylation was increased in the congenic mice (B), while *Klk7* gene ablation itself did not affect the levels of phospho-tau (A) ((*) for $p < 0.05$, (**) for $p < 0.01$ by student *t*-test). (C) Thioflavin S staining (green) of the brains of 3 months of age male *App*$^{NL\text{-}G\text{-}F/NL\text{-}G\text{-}F}$*; Klk7*$^{-/-}$ mice with counter immunostaining using an anti-BACE1 antibody (red). Scale bar, 100 μm. (D) Thioflavin S (Thio S) staining (green) of the brains of 3 months of age male *App*$^{NL\text{-}G\text{-}F/NL\text{-}G\text{-}F}$*; Klk7*$^{-/-}$ mice with counter immunostaining using an anti-GFAP antibody (magenta) and DRAQ5 (blue). Scale bar, 100 μm.

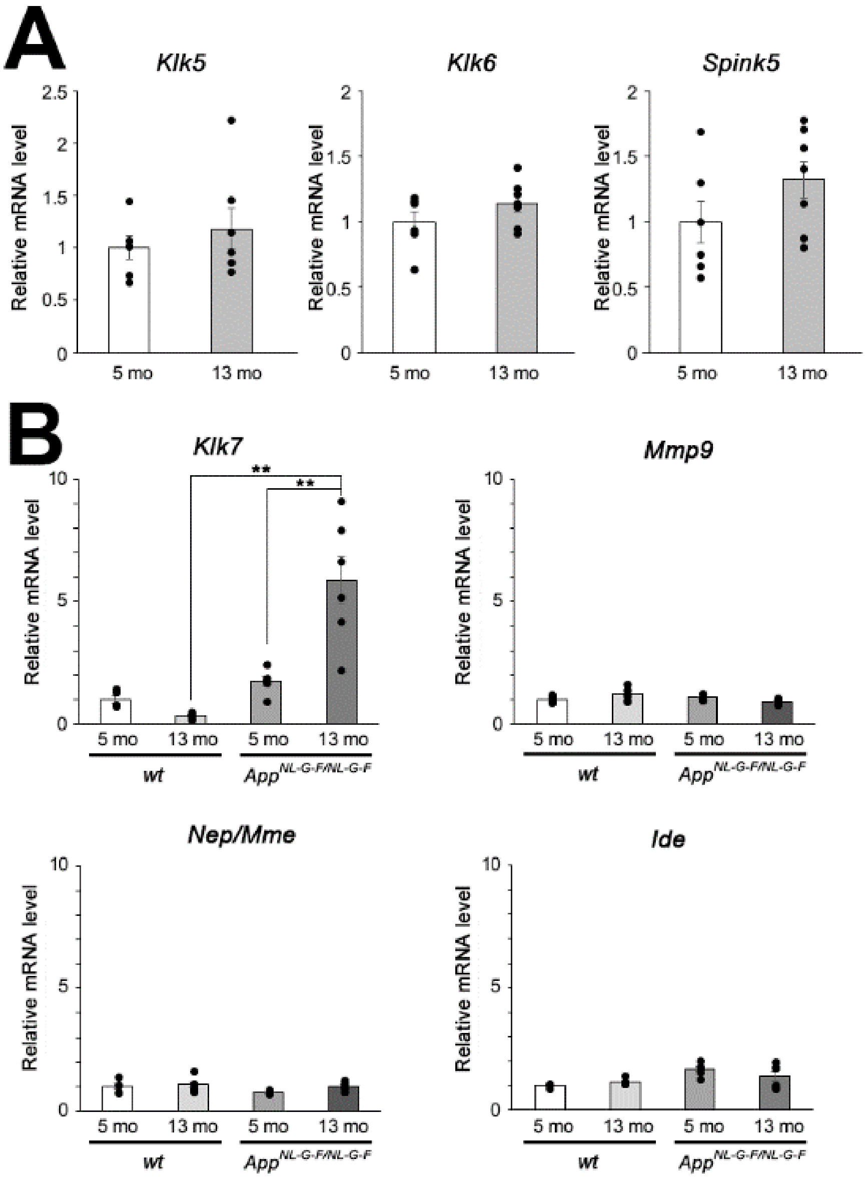

Appendix Fig. S9. Expression levels of mRNAs of Klks and other Aβ degrading enzymes in brains of aged mice

(A) Levels of *Klk5*, *Klk6* and *Spink5* mRNAs in 5 and 13 months of age male *App*$^{NL\text{-}G\text{-}F/NL\text{-}G\text{-}F}$ mice (n = 5 or 6, mean ± s.e.m.) (B) Levels of *Klk7*, *Mmp9*, *Nep/Mme* and *Ide* mRNAs in 5 and 13 months of age male wild-type or *App*$^{NL\text{-}G\text{-}F/NL\text{-}G\text{-}F}$ mice (n = 5 or 6, mean ± s.e.m., (**) for $p < 0.01$ by Tukey's test). Expression levels were standardized by that of 5 months of age wild-type mice.

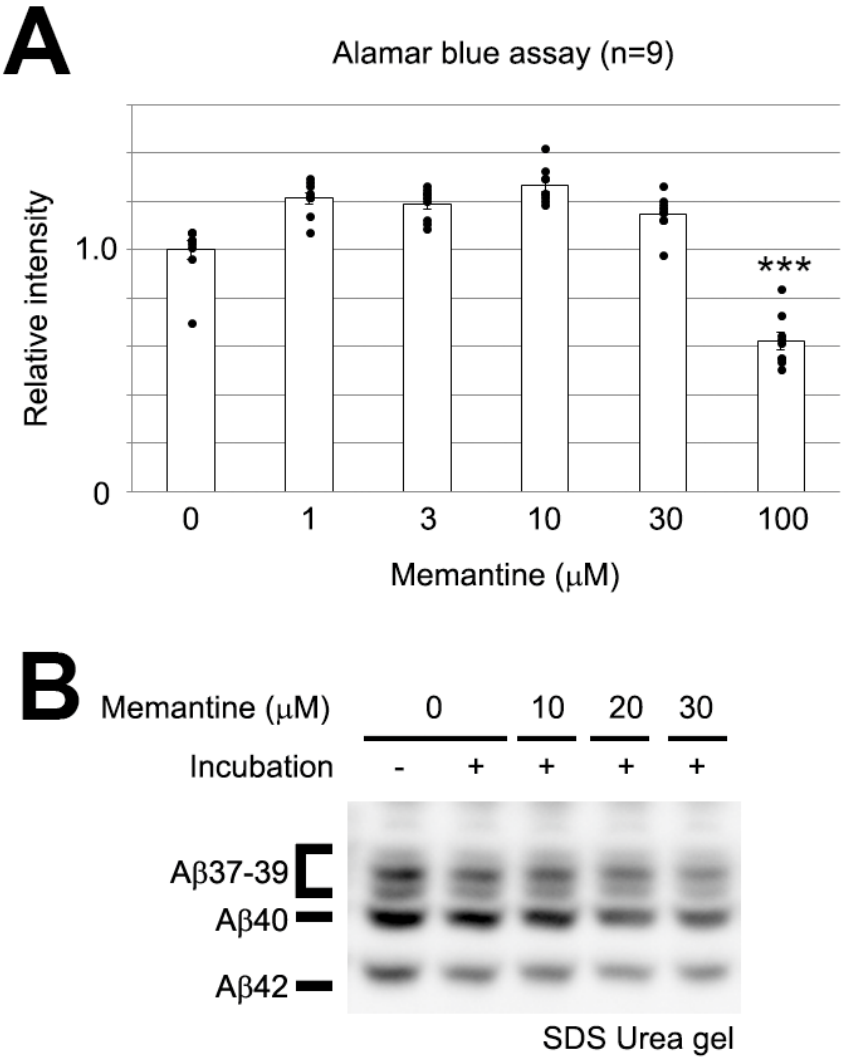

Appendix Fig. S10. Effects of memantine on cell viability and Aβ degradation activity in primary astrocytes

(A) Effect of memantine on cell viability of primary astrocytes analyzed by alamar blue assay (n = 9, mean ± s.e.m., (***) for p < 0.001 by Tukey's test). (B) Aβ degradation activity in the conditioned medium from memantine-treated primary astrocytes. The conditioned medium of 7PA2 cells were used as substrate source.

Figures		p values
Fig. 3A	mock vs MAB2624 (Aβ40)	0.0282173
	mock vs MAB2624 (Aβ42)	0.0170738
Fig. 3C	mock vs KLK7	4.059E-05
	KLK7 vs KLK7+MAB2624	0.0001602
Fig. 3E (MBP-hKLK7)	0 hr vs 24 hr	0.0067665
	0 hr vs 48 hr	0.0010053
	24 hr vs 48 hr	0.0348396
Fig. 4B	mock vs MAB2624 (Aβ40)	0.0029569
	mock vs MAB2624 (Aβ42)	0.0024501
Fig. 5E (amyloid Aβ)	*Klk7+/+* vs *Klk7-/-*	6.936E-05
Fig. 5E (ThioS)	*Klk7+/+* vs *Klk7-/-*	2.73E-06
Fig. 6A	wt vs *NL-G-F/NL-G-F*	0.0023668
Fig. 6B	5 mo vs 13 mo	0.0009525
Fig. 6C	mock vs 50 nM	0.039597
Fig. 7B	mock vs Mem	5.443E-07
Fig. 7C	Veh vs Mem	0.046785
Fig. 7D	mock vs Mem	0.0075283
Fig. 7E	mock vs NMDA	0.0068135
	mock vs L-Glu	0.0010053
Fig7F	*Klk7+/+*	
	mock vs Mem (Aβ40)	0.0017282
	mock vs Mem (Aβ42)	0.0116827
Appendix		
Fig. S1C	0h vs 24 hr mock	0.00004
	24 hr mock vs 24 DIFP	0.0047
Fig. S3A	Aβ40	0.0064
	Aβ42	0.0365
Fig. S3C	KLK7 low	0.00008
	KLK7 middle	0.000002

	KLK7 high	0.000002
Fig. S5E	Female Aβ40	0.0368442
	Female Aβ42	0.0193714
Fig. S5E	male Aβ40	0.0419957
	male Aβ42	0.0098054
Fig. S5G	MBP vs hKLK7	0.0014794
Fig. S6A	Total Aβ	0.000905
	Aβ40	0.0001041
	Aβ42	0.0003313
Fig. S6B	Total Aβ	0.0007027
	Aβ40	0.0005853
	Aβ42	0.0051136
Fig. S6C	Total Aβ	0.0003312
	Aβ40	0.0003342
	Aβ42	0.0029319
Fig. S7B	GFAP	5.696E-05
Fig. S8B	*Klk7+/+ vs Klk7-/-* AT180	0.0002357
	Klk7+/+ vs *Klk7-/-* AT270	0.0046134
Fig. S9 (KLK7)	wt 13 mo vs 13 mo NLGF	0.0010053
	5 mo NLGF vs 13 mo NLGF	0.0010053
Fig. S10A	0 µM vs 100 µM	0.000002

Appendix Table S1. All p values for figures
