# Supplementary figures and images for: Loss of kallikrein‐related peptidase 7 exacerbates amyloid pathology in Alzheimer's disease model mice
Source: EMBO Mol Med. 2018 Jan 8;10(3):e8184. doi: 10.15252/emmm.201708184 (PMC5840542; doi:10.15252/emmm.201708184)

A $\beta$

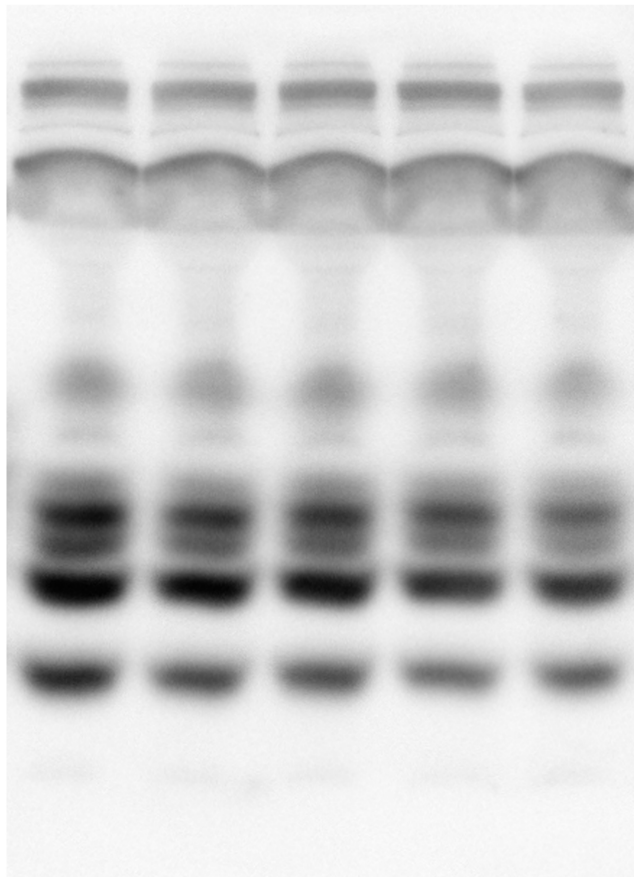

Supplement: Supplementary file 2 — Source Data for Appendix [file EMMM-10-e8184-s006.zip › Source_Data_Appendix_figures/S10B.pdf]

A $\beta$

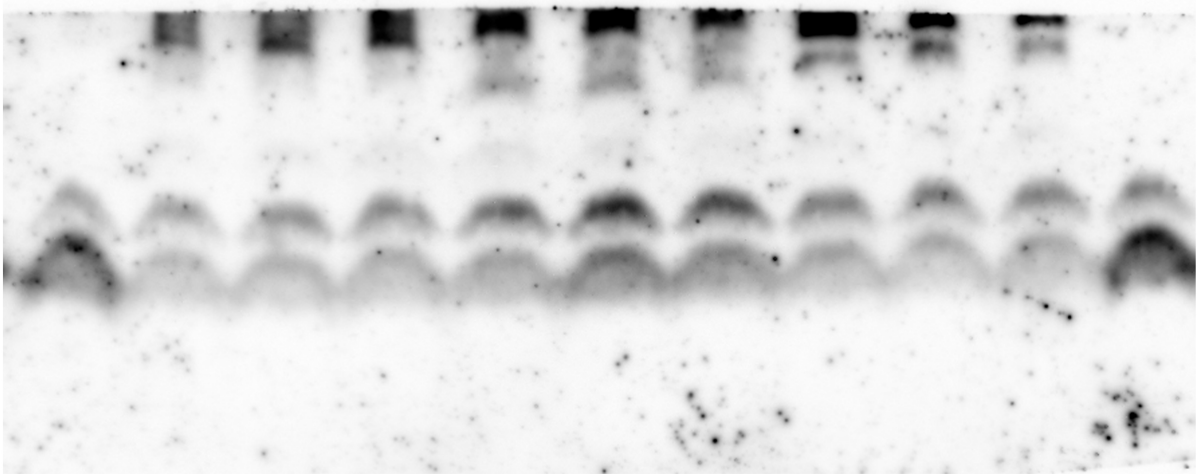

Supplement: Supplementary file 2 — Source Data for Appendix [file EMMM-10-e8184-s006.zip › Source_Data_Appendix_figures/S4B.pdf]

A $\beta$

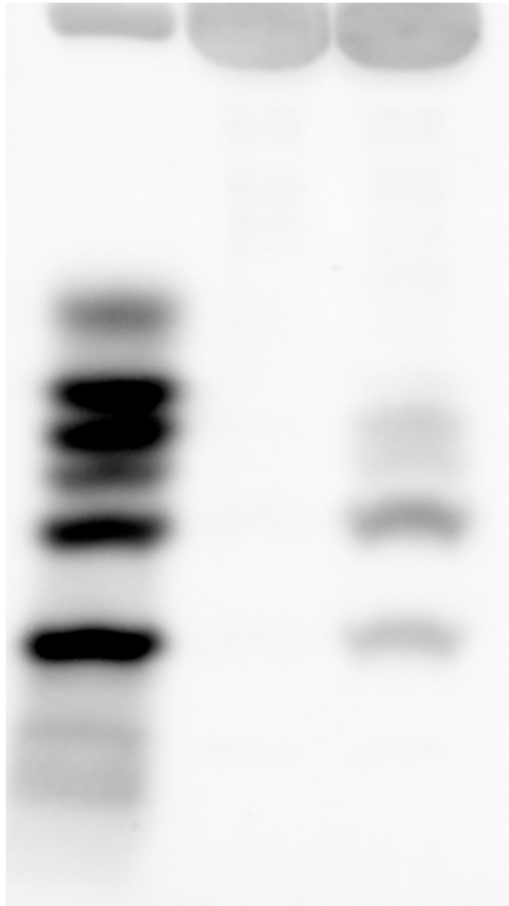

Supplement: Supplementary file 2 — Source Data for Appendix [file EMMM-10-e8184-s006.zip › Source_Data_Appendix_figures/S1/S1A.pdf]

A $\beta$

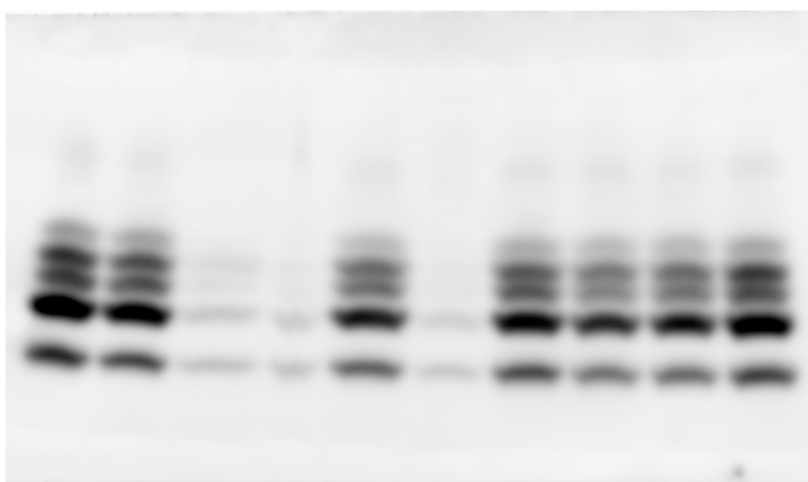

Supplement: Supplementary file 2 — Source Data for Appendix [file EMMM-10-e8184-s006.zip › Source_Data_Appendix_figures/S1/S1B.pdf]

A $\beta$

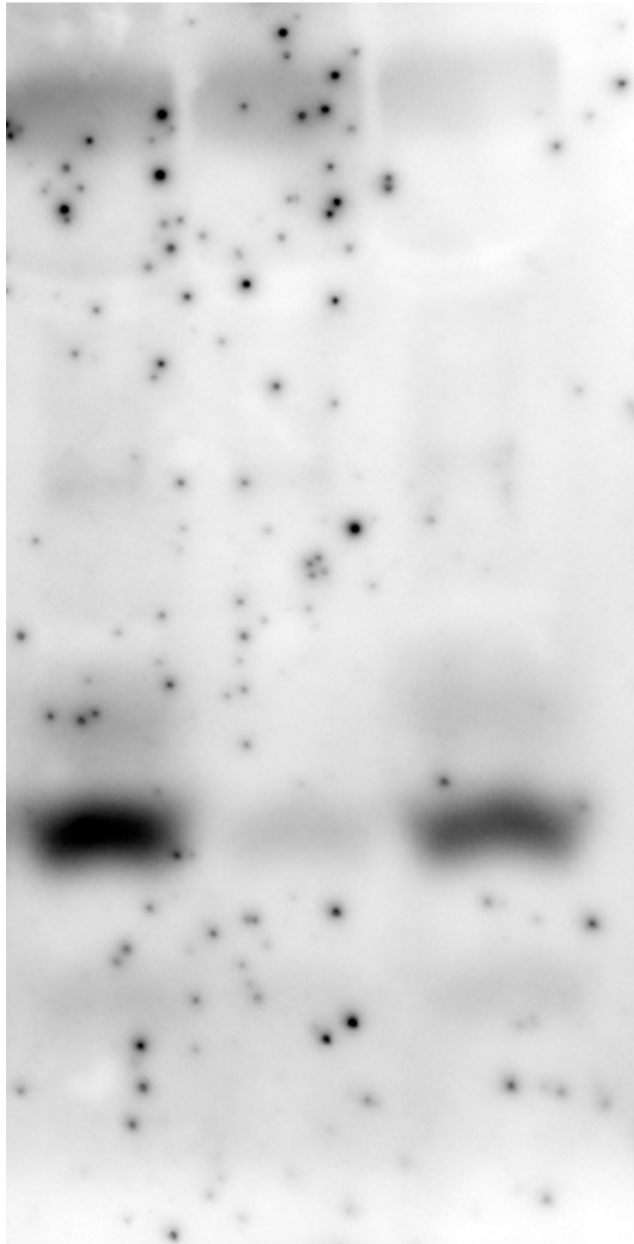

Supplement: Supplementary file 2 — Source Data for Appendix [file EMMM-10-e8184-s006.zip › Source_Data_Appendix_figures/S1/S1C.pdf]

A $\beta$

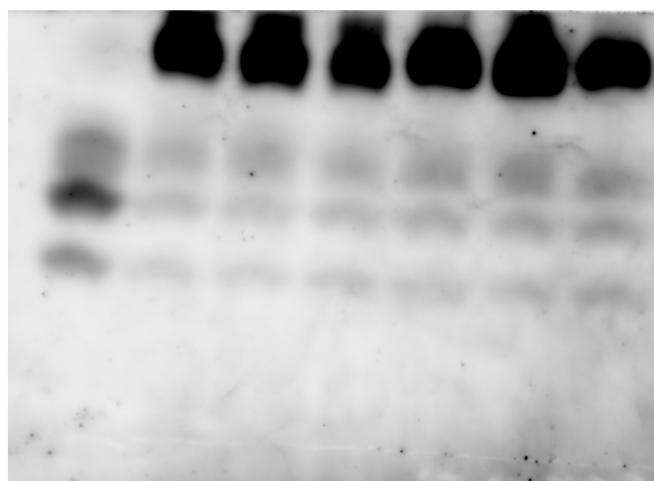

Supplement: Supplementary file 2 — Source Data for Appendix [file EMMM-10-e8184-s006.zip › Source_Data_Appendix_figures/S3/S3A.pdf]

# KLK7

(kDa)

37

25

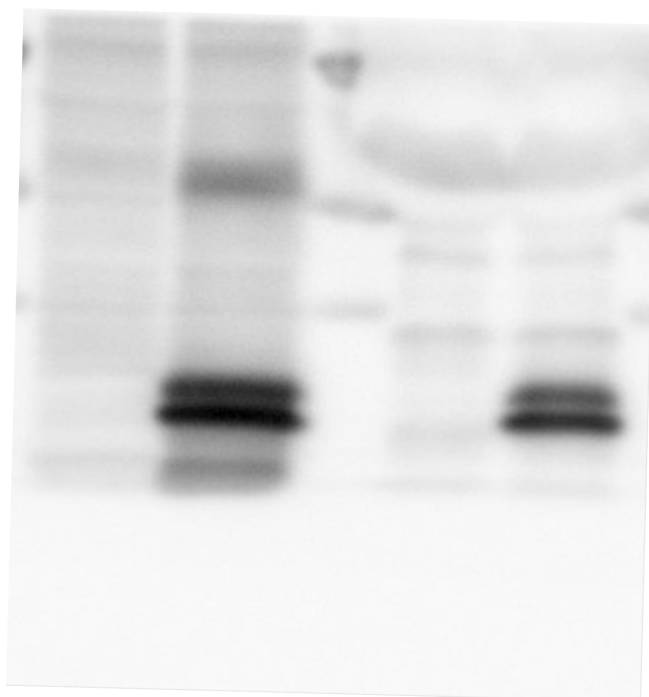

Supplement: Supplementary file 2 — Source Data for Appendix [file EMMM-10-e8184-s006.zip › Source_Data_Appendix_figures/S3/S3B_KLK7.pdf]

$\alpha$ -tubulin

50

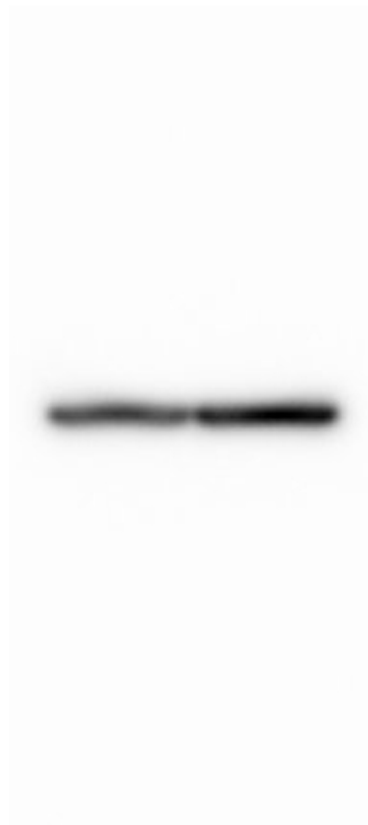

Supplement: Supplementary file 2 — Source Data for Appendix [file EMMM-10-e8184-s006.zip › Source_Data_Appendix_figures/S3/S3B_tubulin.pdf]

MBP

75

50

37

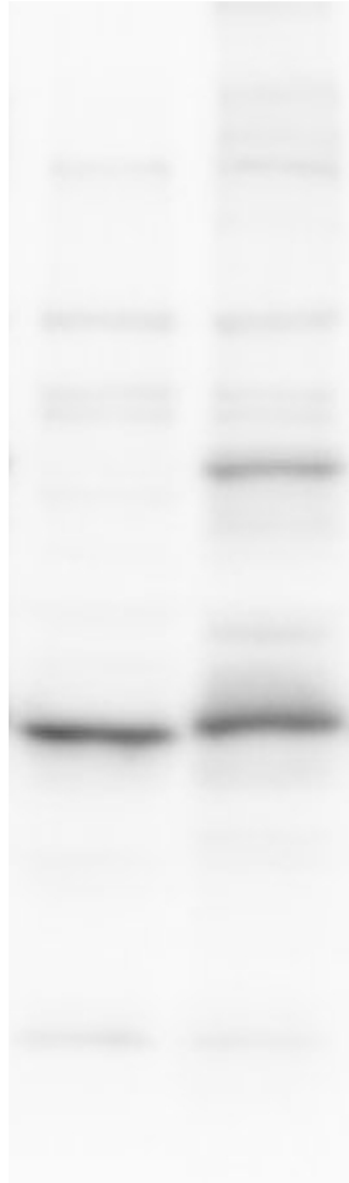

Supplement: Supplementary file 2 — Source Data for Appendix [file EMMM-10-e8184-s006.zip › Source_Data_Appendix_figures/S3/S3D_MBP.pdf]

A $\beta$

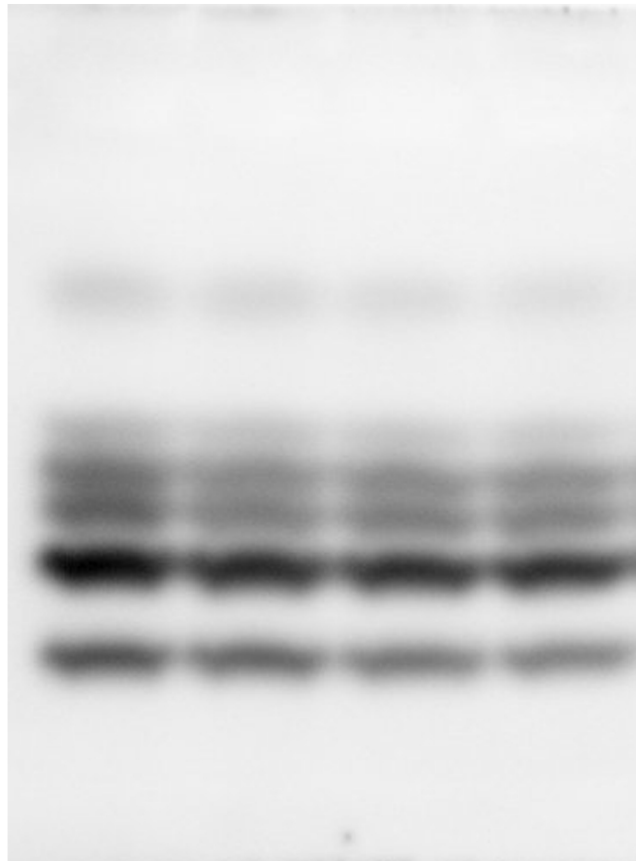

Supplement: Supplementary file 2 — Source Data for Appendix [file EMMM-10-e8184-s006.zip › Source_Data_Appendix_figures/S3/S3E_KLK6.pdf]

A $\beta$

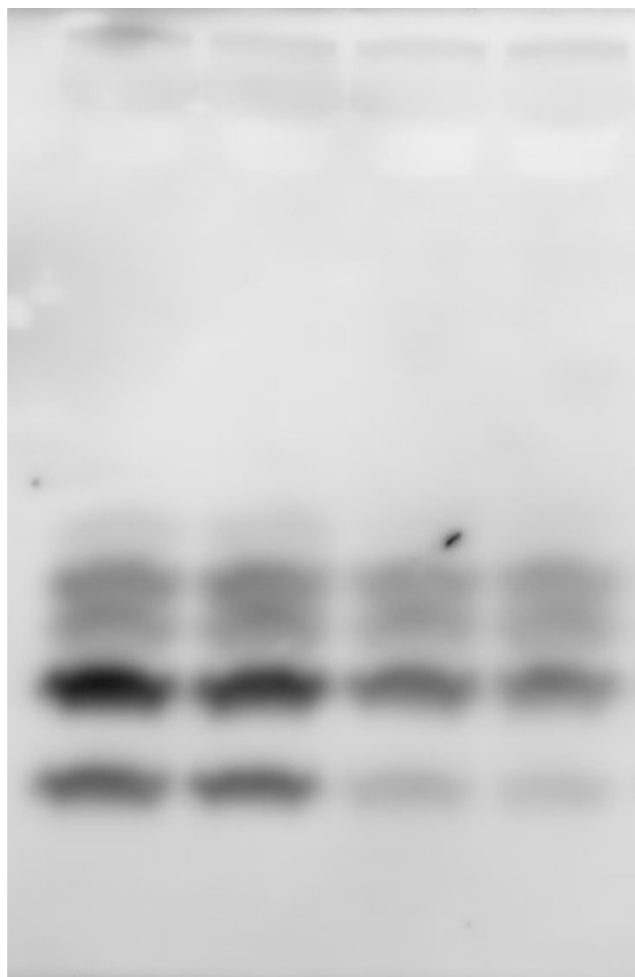

Supplement: Supplementary file 2 — Source Data for Appendix [file EMMM-10-e8184-s006.zip › Source_Data_Appendix_figures/S3/S3E_KLK7.pdf]

A $\beta$

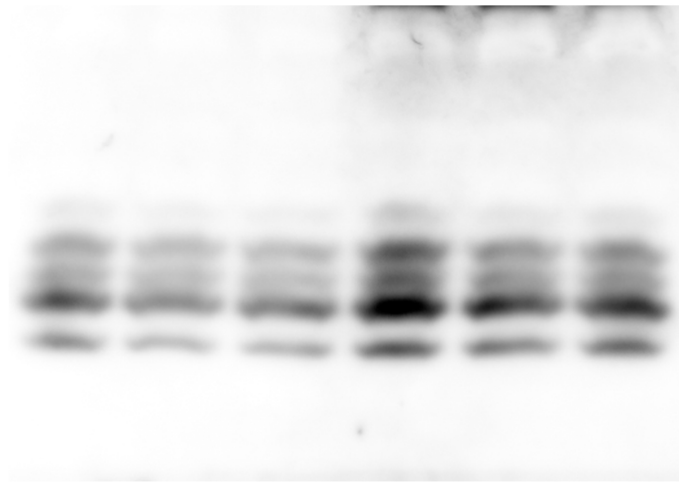

Supplement: Supplementary file 2 — Source Data for Appendix [file EMMM-10-e8184-s006.zip › Source_Data_Appendix_figures/S5/S5D_Klk7++.pdf]

A $\beta$

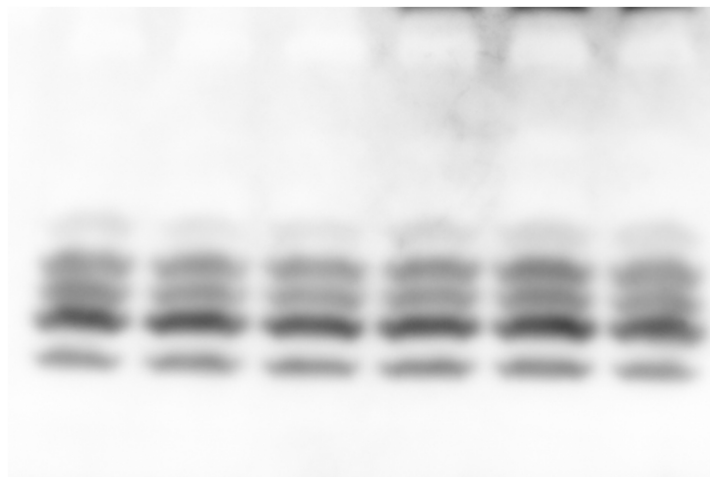

Supplement: Supplementary file 2 — Source Data for Appendix [file EMMM-10-e8184-s006.zip › Source_Data_Appendix_figures/S5/S5D_Klk7--.pdf]

ApoE

37

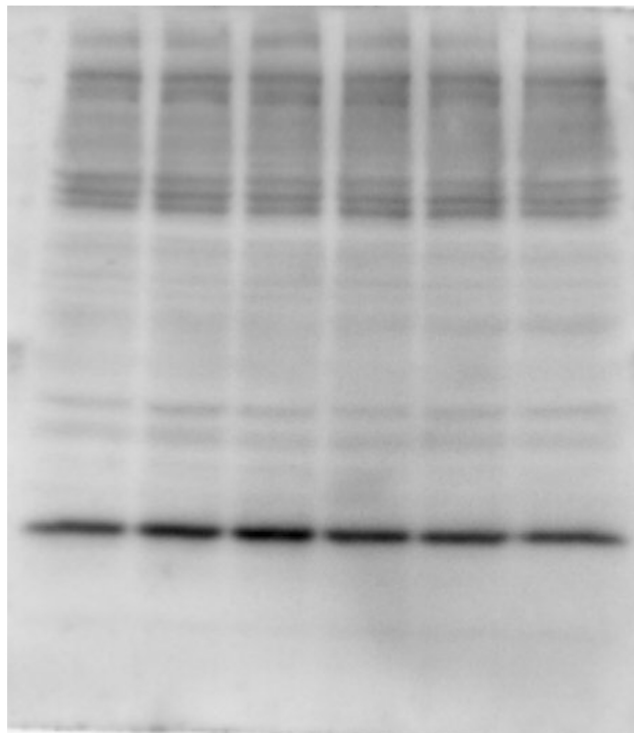

Supplement: Supplementary file 2 — Source Data for Appendix [file EMMM-10-e8184-s006.zip › Source_Data_Appendix_figures/S5/S5F_ApoE.pdf]

# APP-CTF

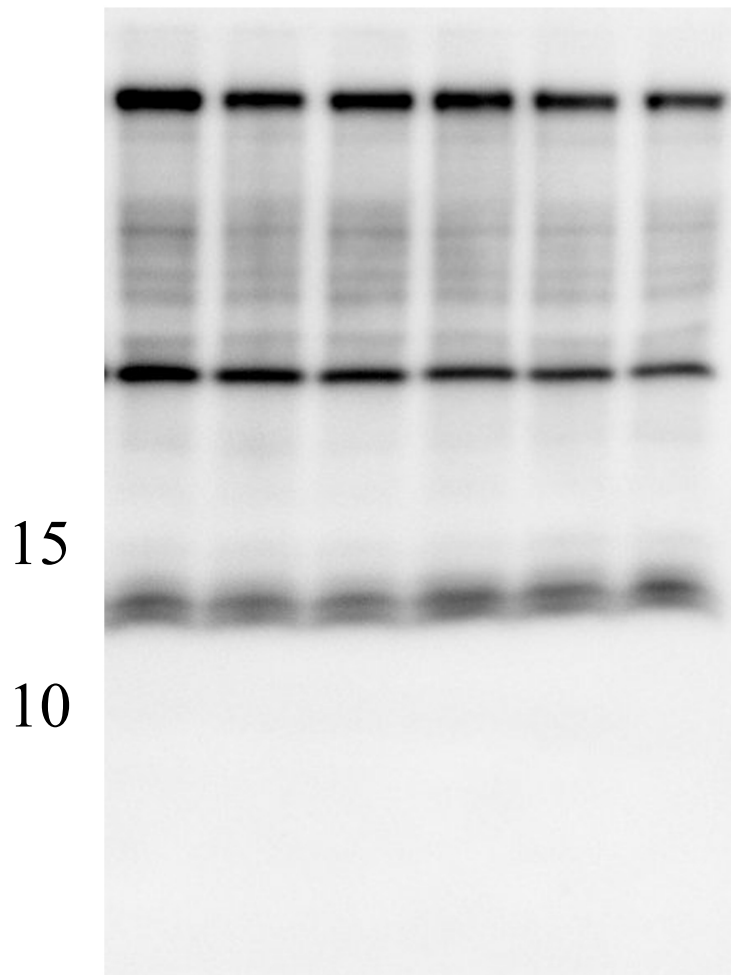

Supplement: Supplementary file 2 — Source Data for Appendix [file EMMM-10-e8184-s006.zip › Source_Data_Appendix_figures/S5/S5F_APP-CTF.pdf]

APP

150

100

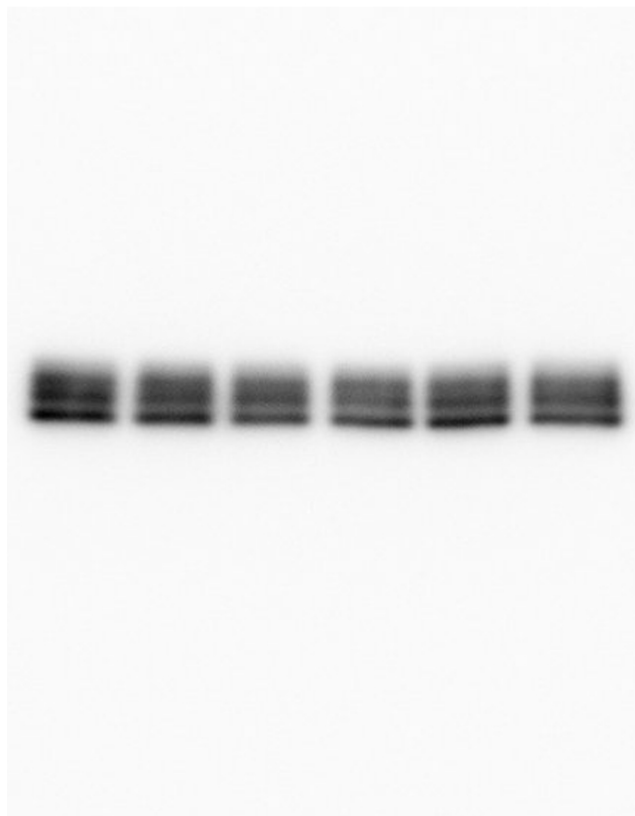

Supplement: Supplementary file 2 — Source Data for Appendix [file EMMM-10-e8184-s006.zip › Source_Data_Appendix_figures/S5/S5F_APP.pdf]

# BACE

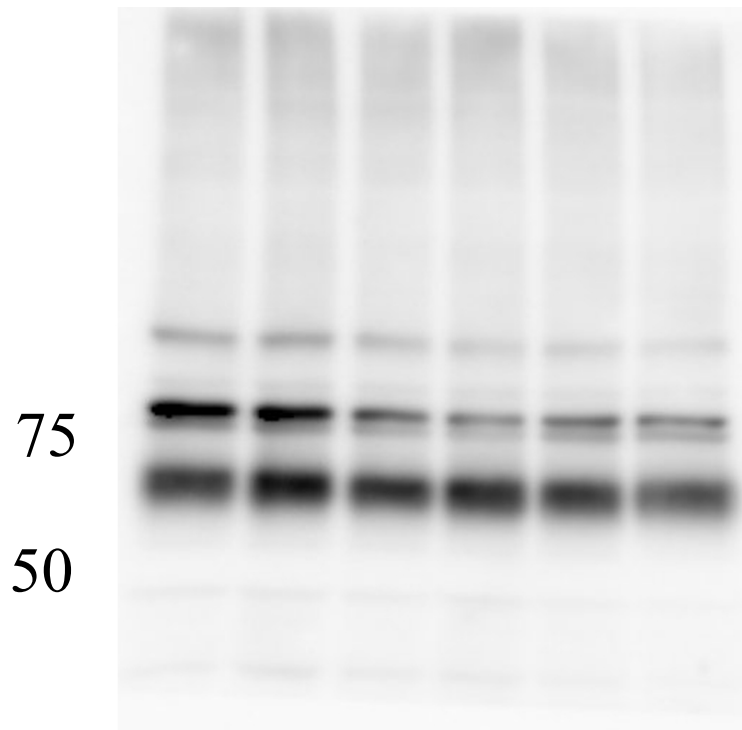

Supplement: Supplementary file 2 — Source Data for Appendix [file EMMM-10-e8184-s006.zip › Source_Data_Appendix_figures/S5/S5F_BACE.pdf]

# IDE

150

100

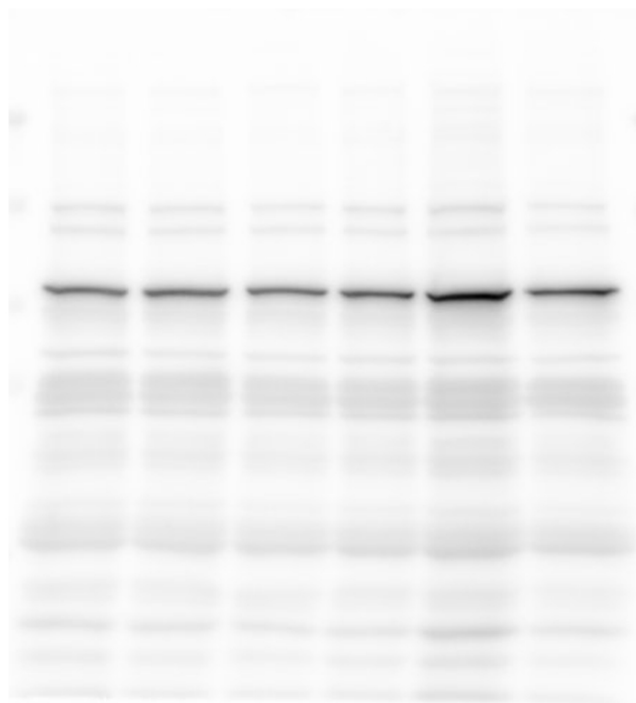

Supplement: Supplementary file 2 — Source Data for Appendix [file EMMM-10-e8184-s006.zip › Source_Data_Appendix_figures/S5/S5F_IDE.pdf]

## MMP-9

100

75

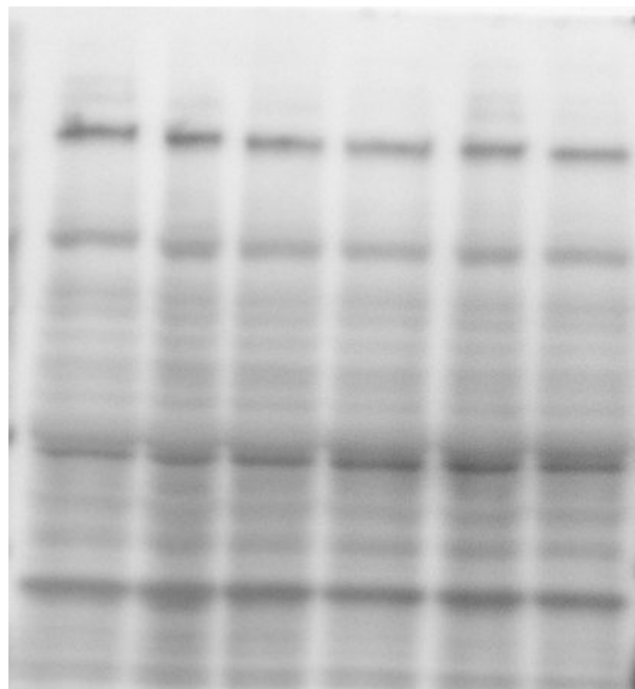

Supplement: Supplementary file 2 — Source Data for Appendix [file EMMM-10-e8184-s006.zip › Source_Data_Appendix_figures/S5/S5F_MMP-9.pdf]

Nct

150

100

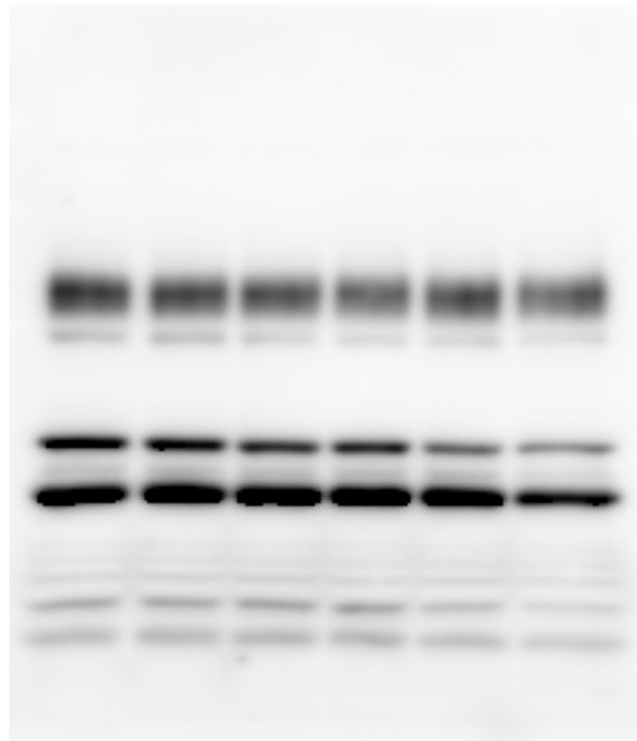

Supplement: Supplementary file 2 — Source Data for Appendix [file EMMM-10-e8184-s006.zip › Source_Data_Appendix_figures/S5/S5F_Nct_corrected.pdf]

NEP

100  
75

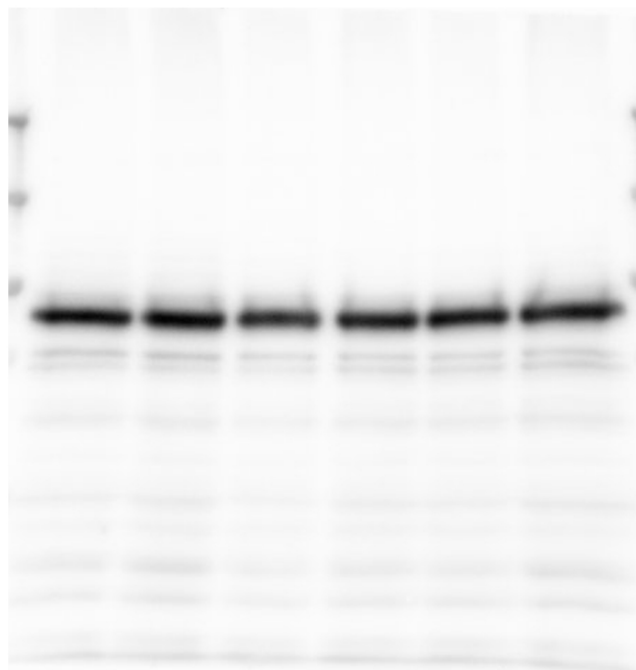

Supplement: Supplementary file 2 — Source Data for Appendix [file EMMM-10-e8184-s006.zip › Source_Data_Appendix_figures/S5/S5F_NEP.pdf]

$\alpha$ -tubulin

50

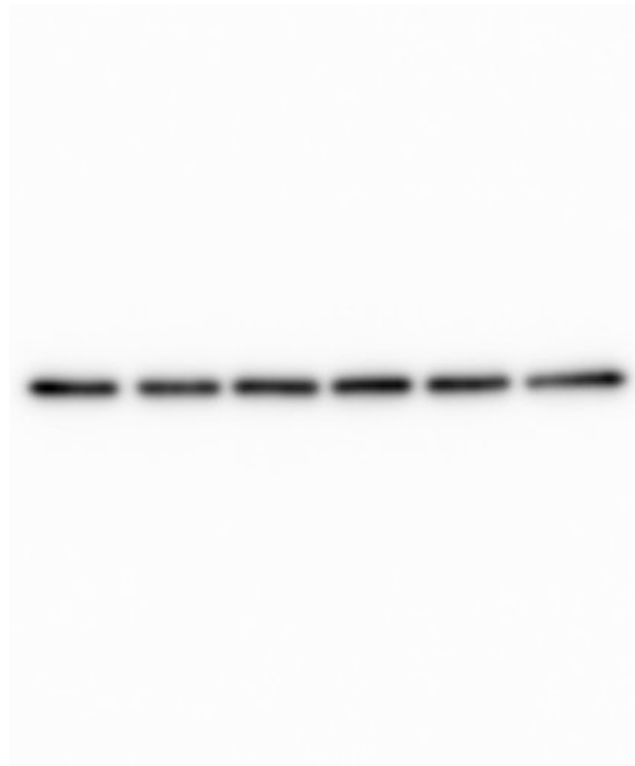

Supplement: Supplementary file 2 — Source Data for Appendix [file EMMM-10-e8184-s006.zip › Source_Data_Appendix_figures/S5/S5F_tubulin.pdf]

BACE

75

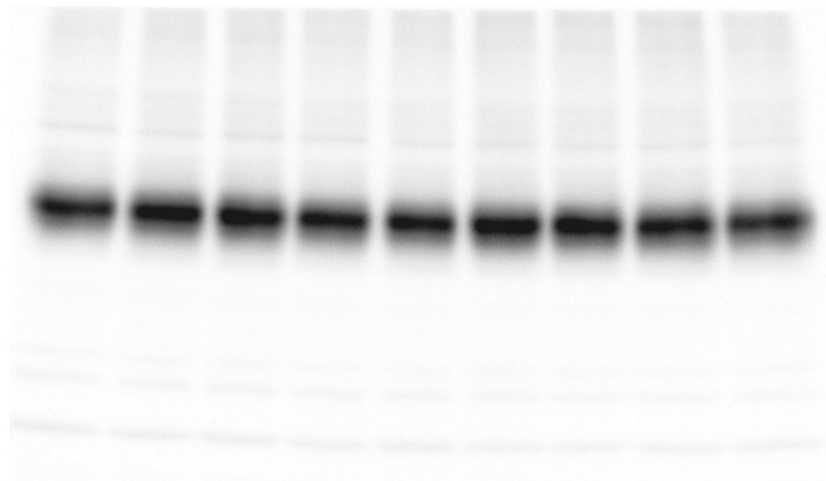

Supplement: Supplementary file 2 — Source Data for Appendix [file EMMM-10-e8184-s006.zip › Source_Data_Appendix_figures/S7/S7__BACE.pdf]

## ADAM10

75

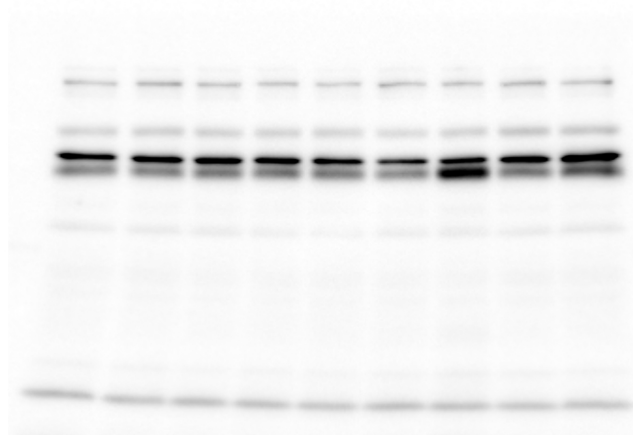

Supplement: Supplementary file 2 — Source Data for Appendix [file EMMM-10-e8184-s006.zip › Source_Data_Appendix_figures/S7/S7_ADAM10.pdf]

# Aldh1L1

(kDa)

100

75

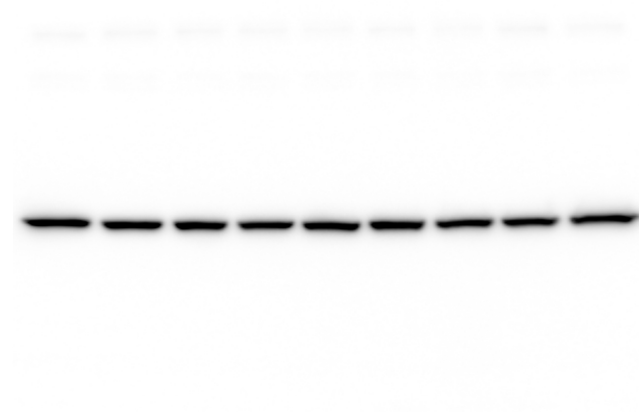

Supplement: Supplementary file 2 — Source Data for Appendix [file EMMM-10-e8184-s006.zip › Source_Data_Appendix_figures/S7/S7_Aldh1L1.pdf]

ApoE

37

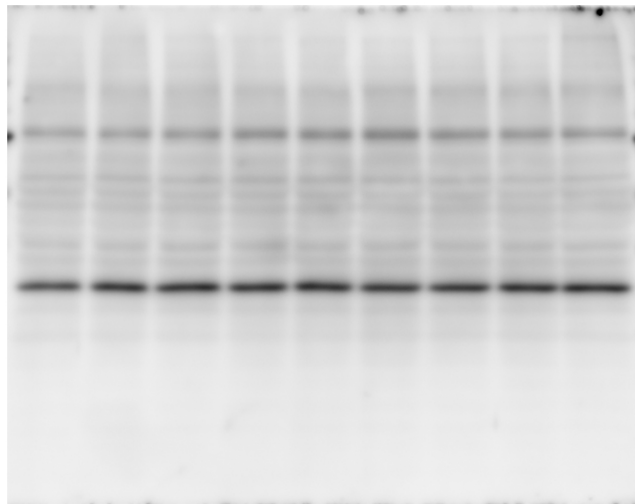

Supplement: Supplementary file 2 — Source Data for Appendix [file EMMM-10-e8184-s006.zip › Source_Data_Appendix_figures/S7/S7_ApoE.pdf]

APP-CTF

10

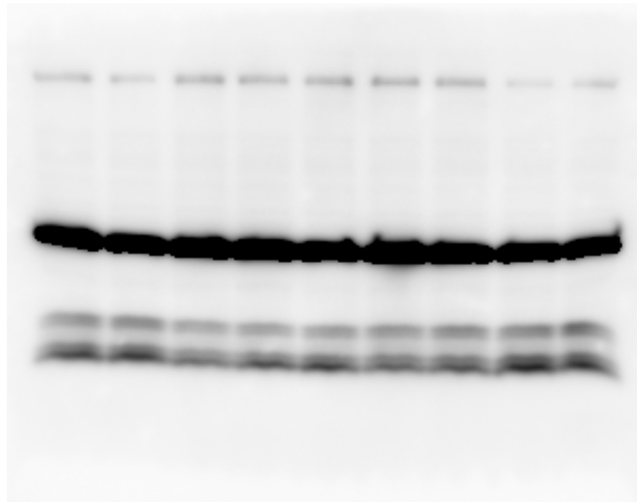

Supplement: Supplementary file 2 — Source Data for Appendix [file EMMM-10-e8184-s006.zip › Source_Data_Appendix_figures/S7/S7_APP-CTF.pdf]

APP

100

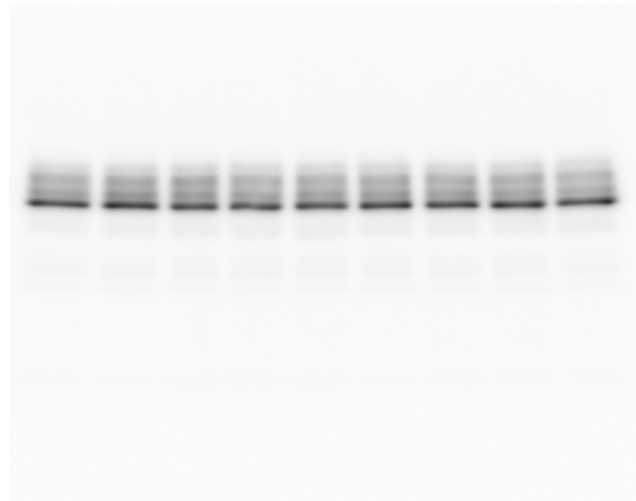

Supplement: Supplementary file 2 — Source Data for Appendix [file EMMM-10-e8184-s006.zip › Source_Data_Appendix_figures/S7/S7_APP.pdf]

IDE

100

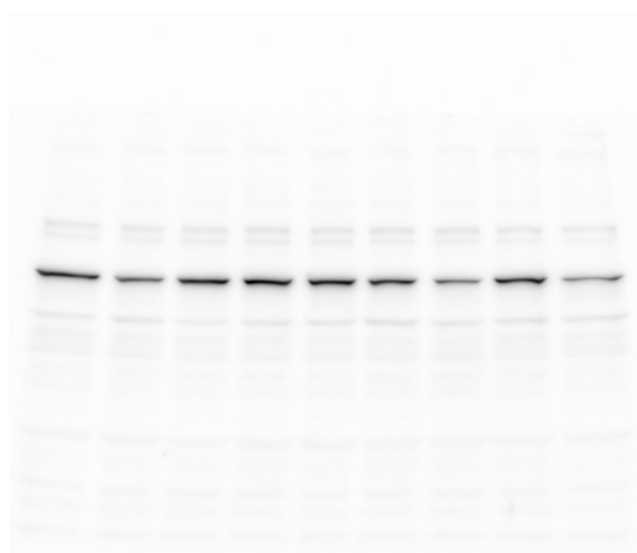

Supplement: Supplementary file 2 — Source Data for Appendix [file EMMM-10-e8184-s006.zip › Source_Data_Appendix_figures/S7/S7_IDE.pdf]

MMP-9

100  
75

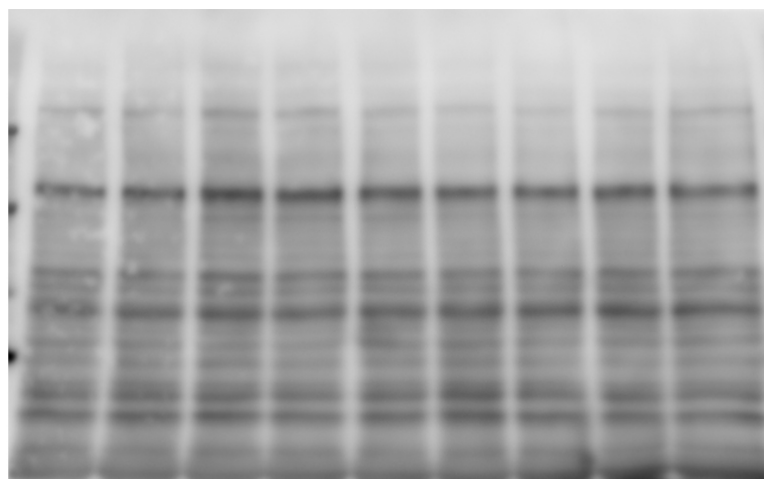

Supplement: Supplementary file 2 — Source Data for Appendix [file EMMM-10-e8184-s006.zip › Source_Data_Appendix_figures/S7/S7_MMP-9_corrected.pdf]

Nct

100

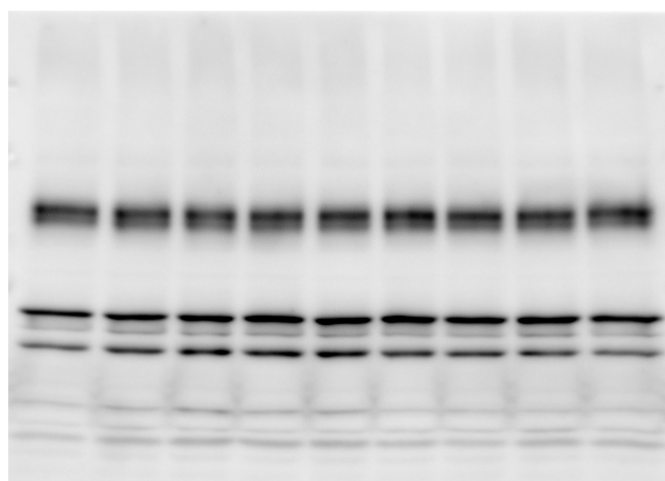

Supplement: Supplementary file 2 — Source Data for Appendix [file EMMM-10-e8184-s006.zip › Source_Data_Appendix_figures/S7/S7_Nct.pdf]

NEP

100

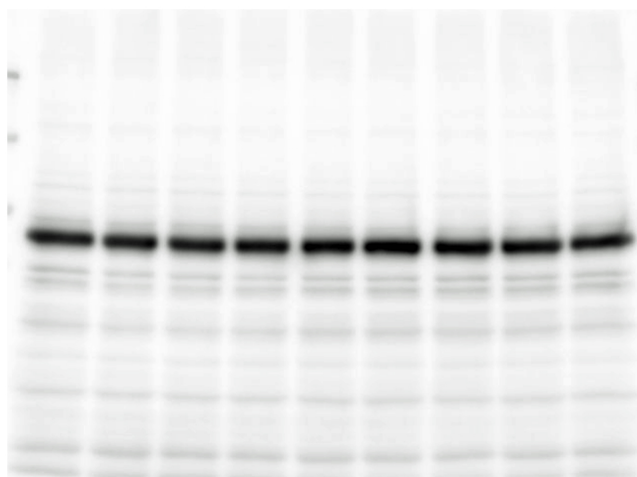

Supplement: Supplementary file 2 — Source Data for Appendix [file EMMM-10-e8184-s006.zip › Source_Data_Appendix_figures/S7/S7_NEP.pdf]

$\alpha$ -tubulin

50

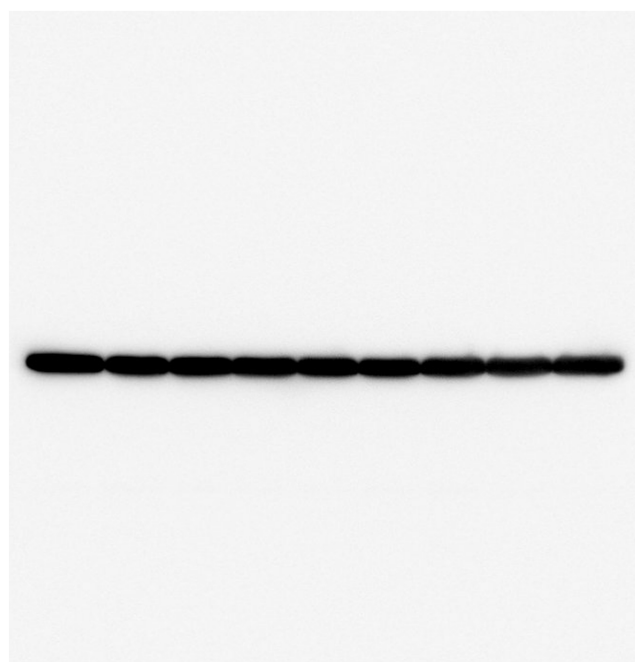

Supplement: Supplementary file 2 — Source Data for Appendix [file EMMM-10-e8184-s006.zip › Source_Data_Appendix_figures/S7/S7_tubulin.pdf]

AT180

50

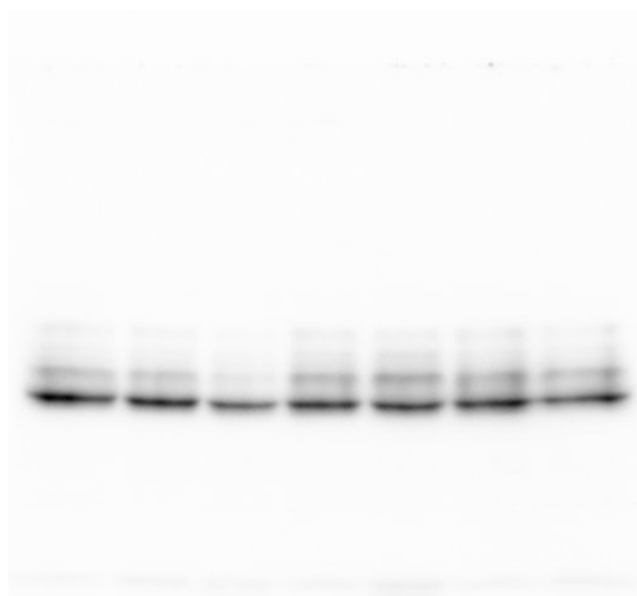

Supplement: Supplementary file 2 — Source Data for Appendix [file EMMM-10-e8184-s006.zip › Source_Data_Appendix_figures/S8/S8A_AT180.pdf]

AT270

50

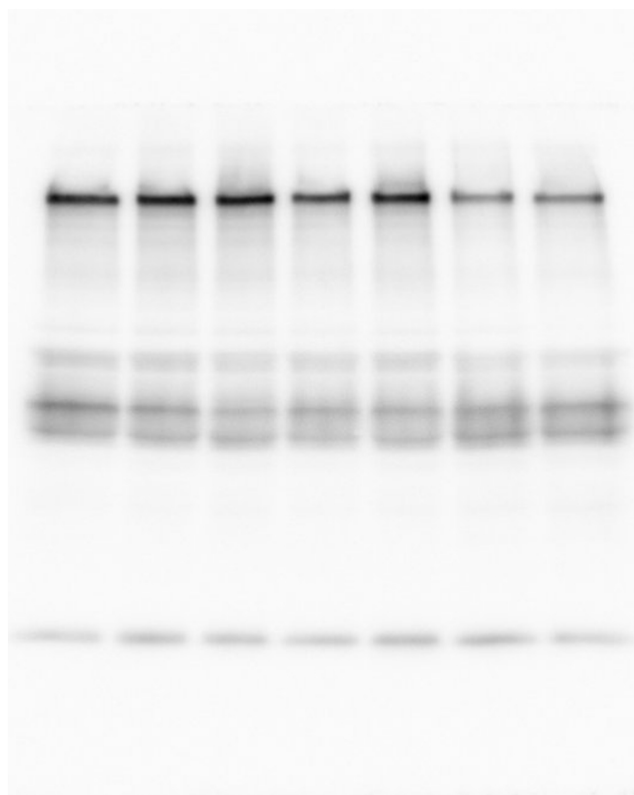

Supplement: Supplementary file 2 — Source Data for Appendix [file EMMM-10-e8184-s006.zip › Source_Data_Appendix_figures/S8/S8A_AT270.pdf]

Tau5

50

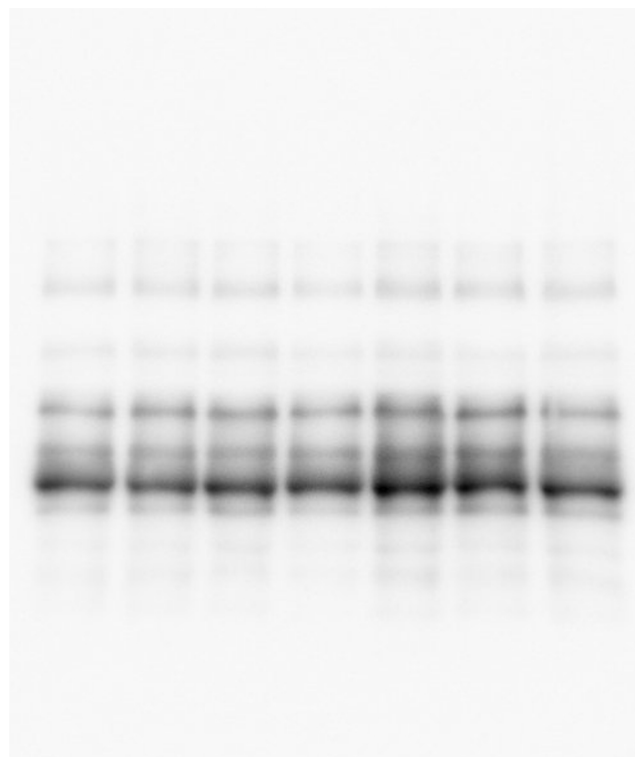

Supplement: Supplementary file 2 — Source Data for Appendix [file EMMM-10-e8184-s006.zip › Source_Data_Appendix_figures/S8/S8A_Tau5.pdf]

$\alpha$ -tubulin

50

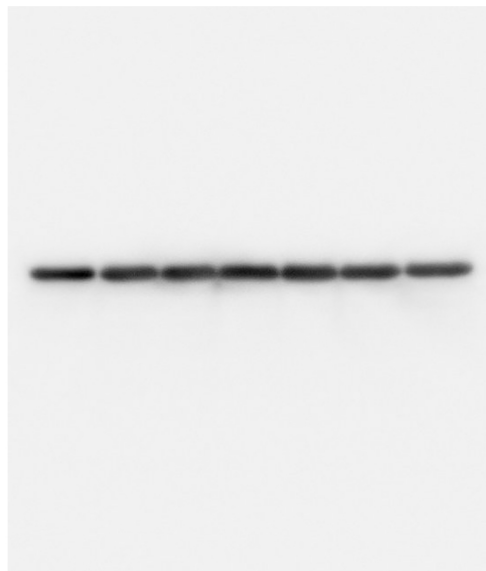

Supplement: Supplementary file 2 — Source Data for Appendix [file EMMM-10-e8184-s006.zip › Source_Data_Appendix_figures/S8/S8A_tubulin_corrected.pdf]

AT180

50

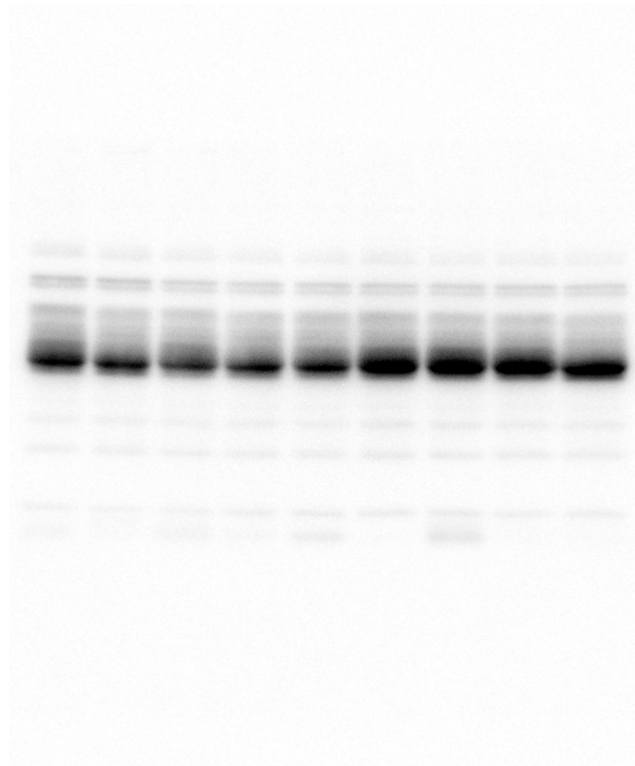

Supplement: Supplementary file 2 — Source Data for Appendix [file EMMM-10-e8184-s006.zip › Source_Data_Appendix_figures/S8/S8B_AT180.pdf]

AT270

50

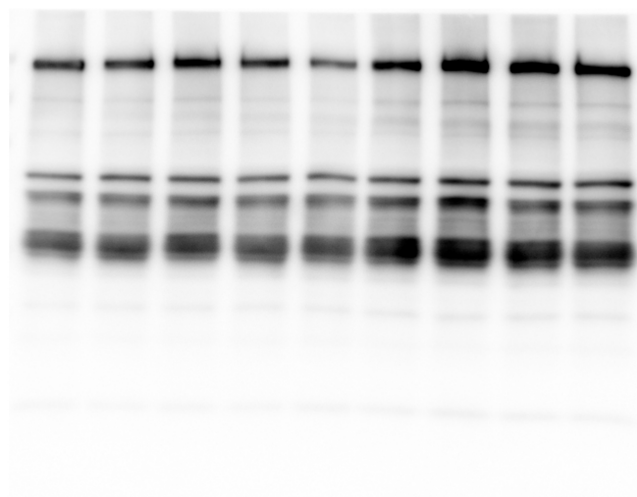

Supplement: Supplementary file 2 — Source Data for Appendix [file EMMM-10-e8184-s006.zip › Source_Data_Appendix_figures/S8/S8B_AT270.pdf]

Tau5

50

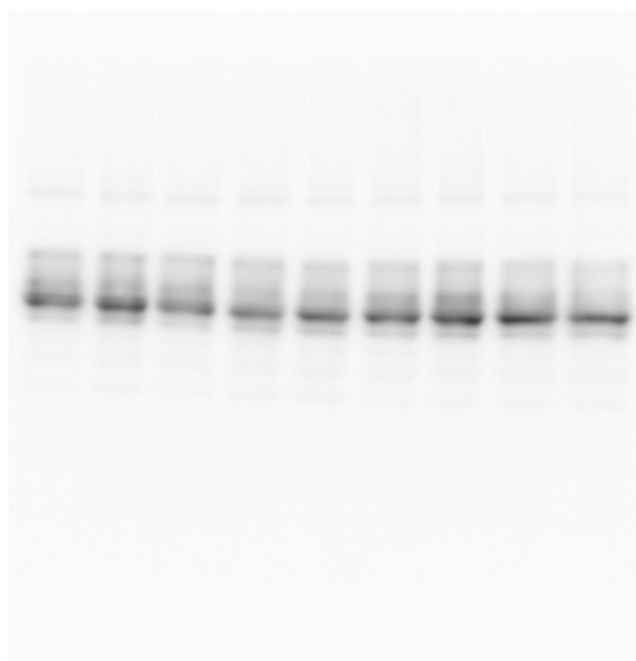

Supplement: Supplementary file 2 — Source Data for Appendix [file EMMM-10-e8184-s006.zip › Source_Data_Appendix_figures/S8/S8B_Tau5.pdf]

$\alpha$ -tubulin

50

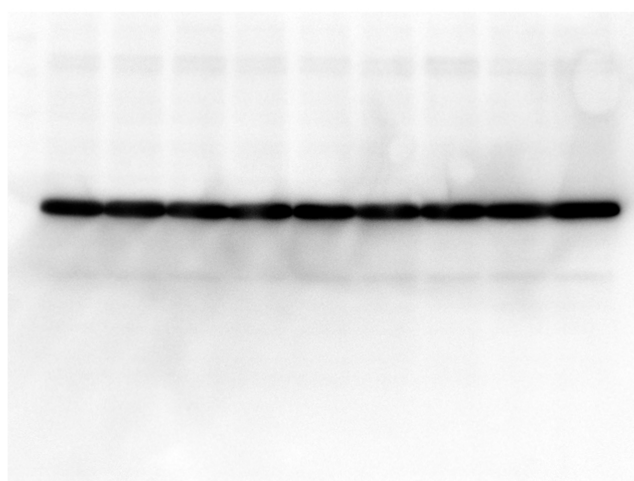

Supplement: Supplementary file 2 — Source Data for Appendix [file EMMM-10-e8184-s006.zip › Source_Data_Appendix_figures/S8/S8B_tubulin.pdf]

A $\beta$

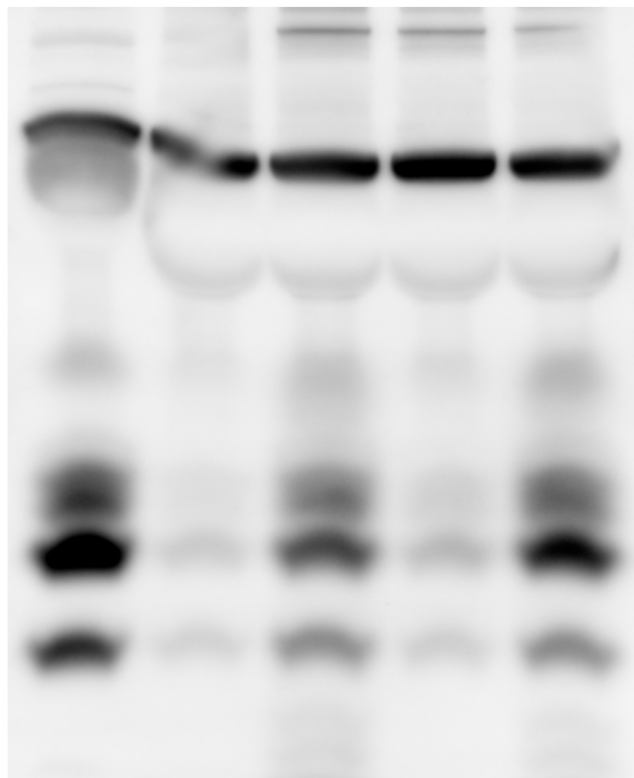

Supplement: Supplementary file 4 — Source Data for Figure 1 [file EMMM-10-e8184-s002.zip › Fig1A.pdf]

A $\beta$

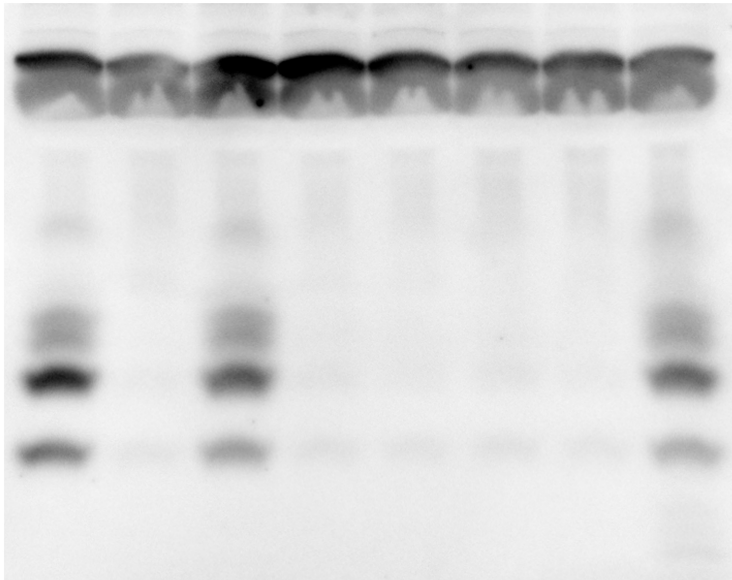

Supplement: Supplementary file 4 — Source Data for Figure 1 [file EMMM-10-e8184-s002.zip › Fig1B.pdf]

A $\beta$

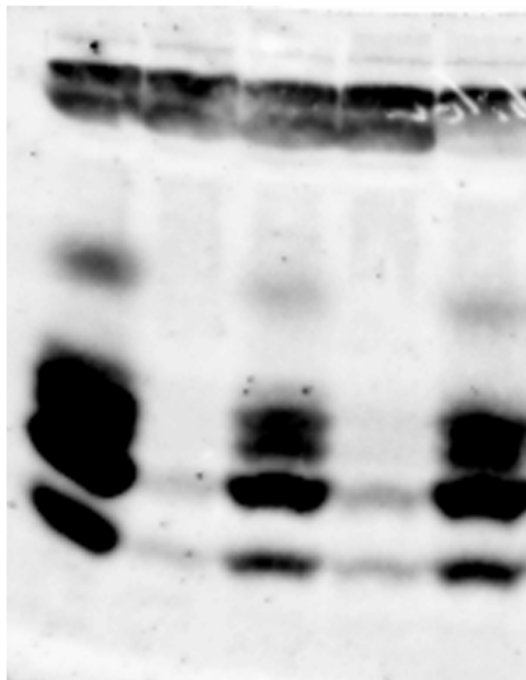

Supplement: Supplementary file 4 — Source Data for Figure 1 [file EMMM-10-e8184-s002.zip › Fig1C.pdf]

A $\beta$

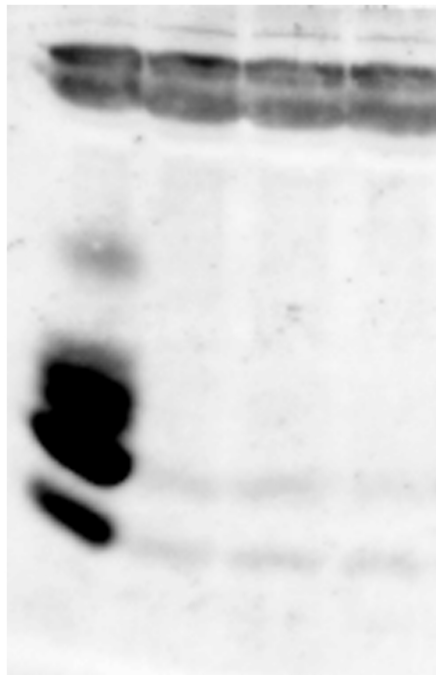

Supplement: Supplementary file 4 — Source Data for Figure 1 [file EMMM-10-e8184-s002.zip › Fig1E.pdf]

A $\beta$

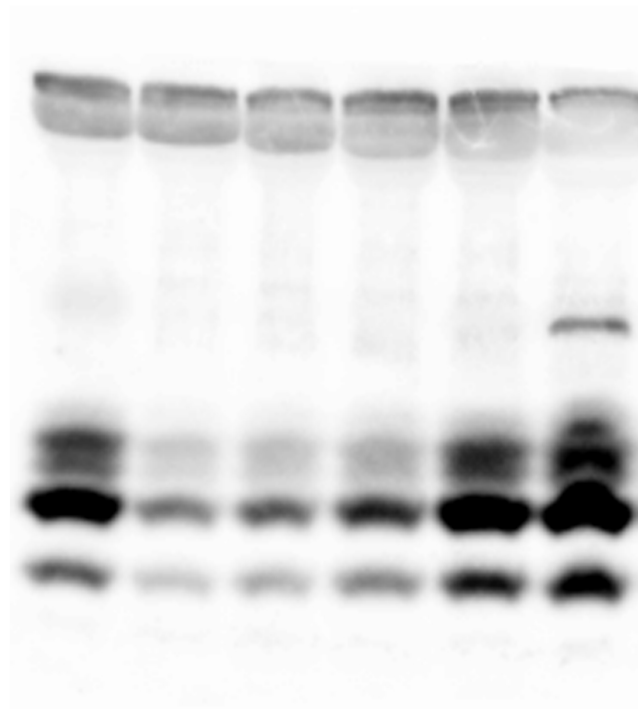

Supplement: Supplementary file 4 — Source Data for Figure 1 [file EMMM-10-e8184-s002.zip › Fig1F.pdf]

A $\beta$

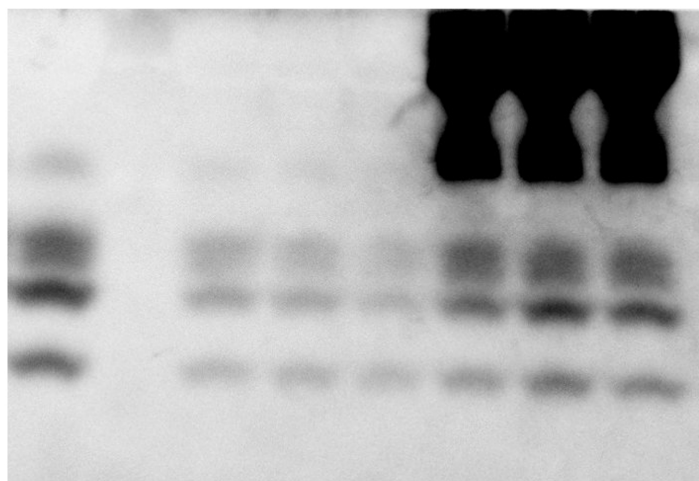

Supplement: Supplementary file 5 — Source Data for Figure 3 [file EMMM-10-e8184-s003.zip › Source_Data_F3/Fig3A.pdf]

A $\beta$

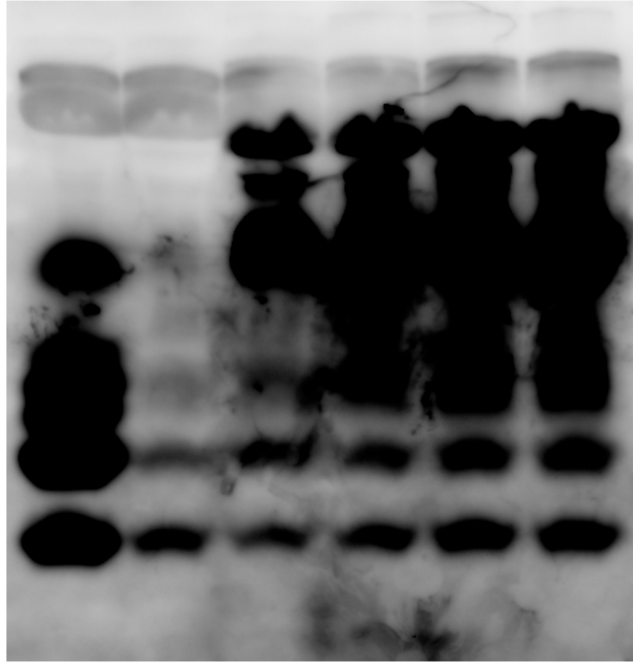

Supplement: Supplementary file 5 — Source Data for Figure 3 [file EMMM-10-e8184-s003.zip › Source_Data_F3/Fig3B.pdf]

A $\beta$

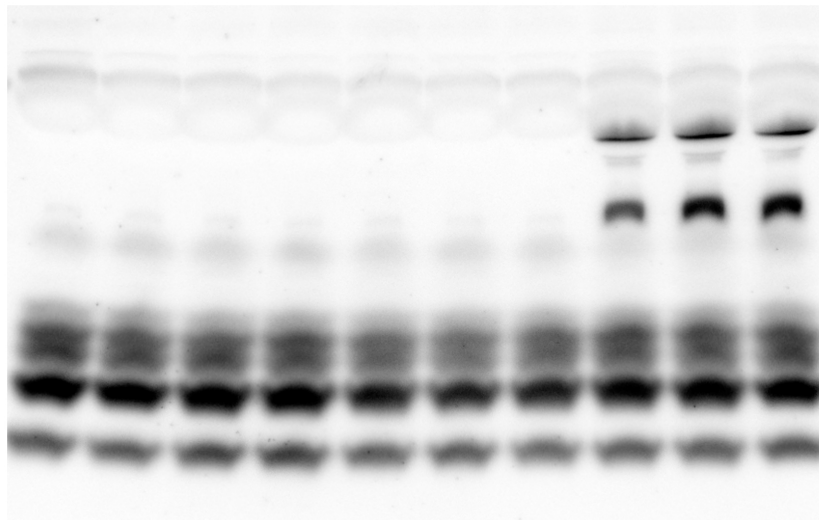

Supplement: Supplementary file 5 — Source Data for Figure 3 [file EMMM-10-e8184-s003.zip › Source_Data_F3/Fig3C.pdf]

A $\beta$

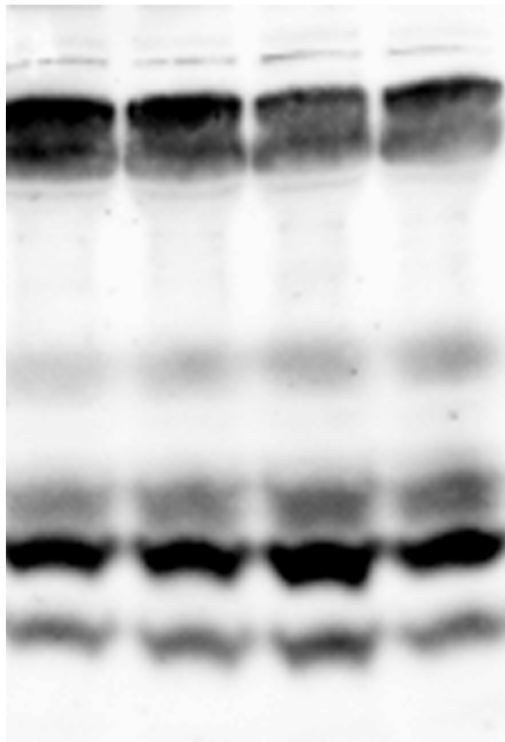

Supplement: Supplementary file 5 — Source Data for Figure 3 [file EMMM-10-e8184-s003.zip › Source_Data_F3/Fig3D_KLK6.pdf]

A $\beta$

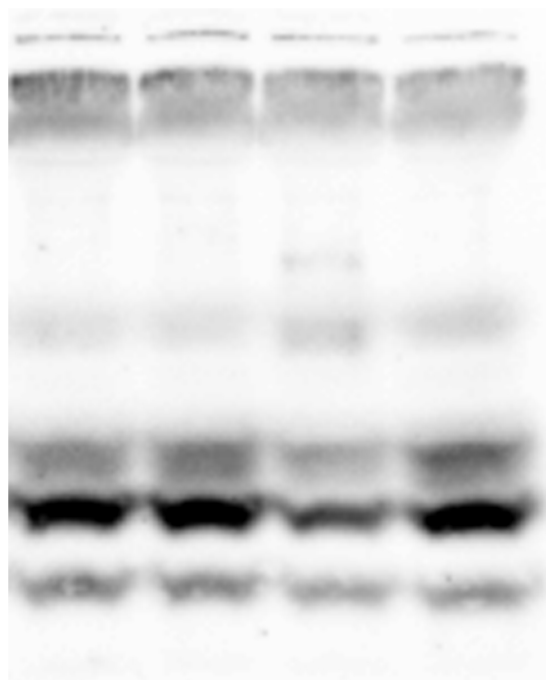

Supplement: Supplementary file 5 — Source Data for Figure 3 [file EMMM-10-e8184-s003.zip › Source_Data_F3/Fig3D_KLK7.pdf]

A $\beta$

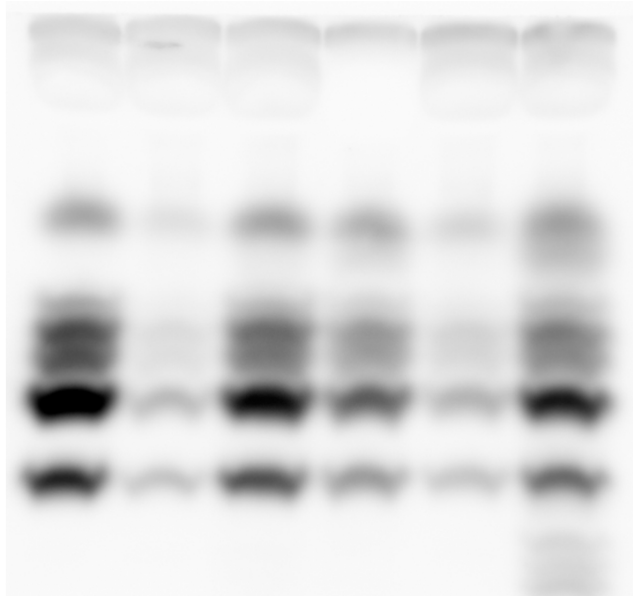

Supplement: Supplementary file 6 — Source Data for Figure 4 [file EMMM-10-e8184-s004.zip › Fig4A.pdf]

A $\beta$

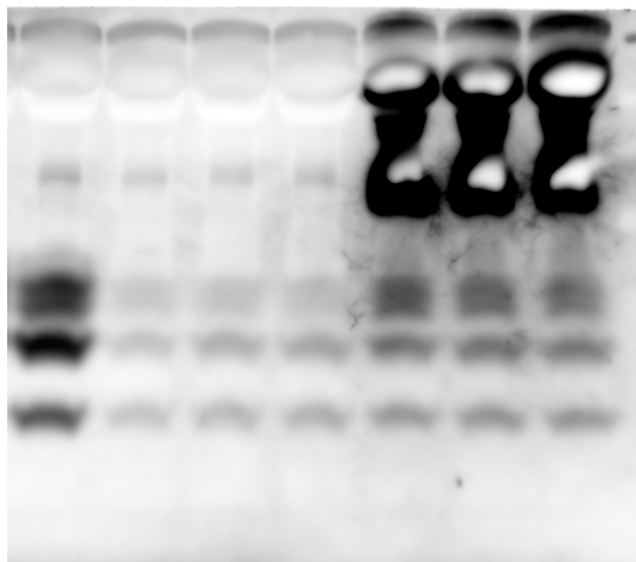

Supplement: Supplementary file 6 — Source Data for Figure 4 [file EMMM-10-e8184-s004.zip › Fig4B.pdf]

A $\beta$

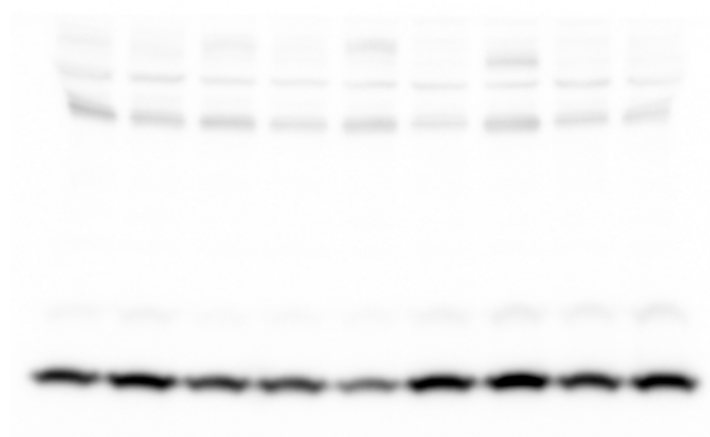

Supplement: Supplementary file 7 — Source Data for Figure 5 [file EMMM-10-e8184-s005.zip › Source_Data_F5/Fig5A-Urea.pdf]

A $\beta$

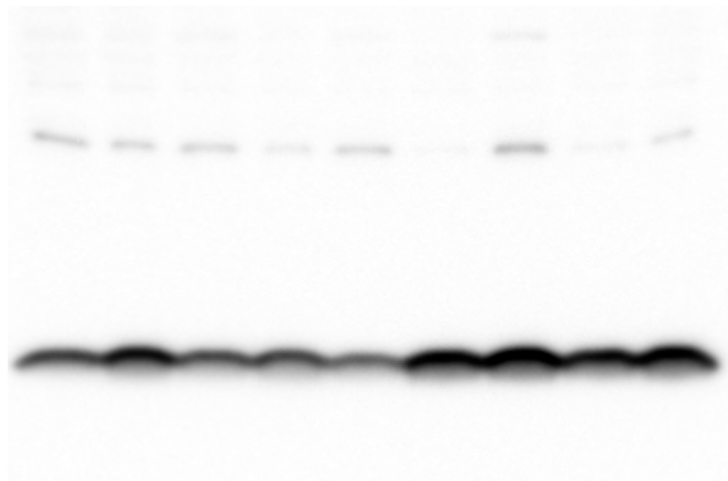

Supplement: Supplementary file 7 — Source Data for Figure 5 [file EMMM-10-e8184-s005.zip › Source_Data_F5/Fig5A_TT.pdf]

A $\beta$

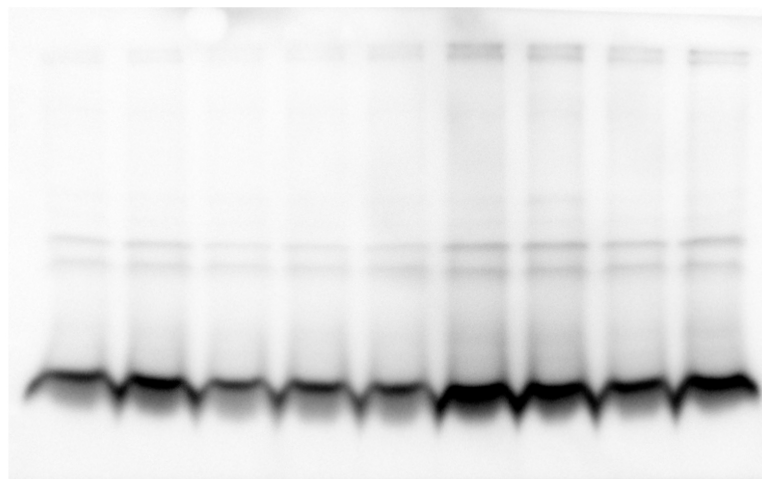

Supplement: Supplementary file 7 — Source Data for Figure 5 [file EMMM-10-e8184-s005.zip › Source_Data_F5/Fig5B_TT.pdf]

A $\beta$

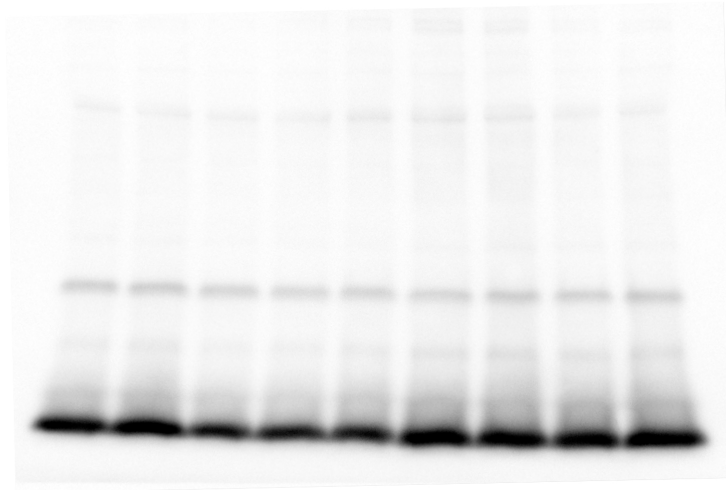

Supplement: Supplementary file 7 — Source Data for Figure 5 [file EMMM-10-e8184-s005.zip › Source_Data_F5/Fig5B_Urea.pdf]

A $\beta$

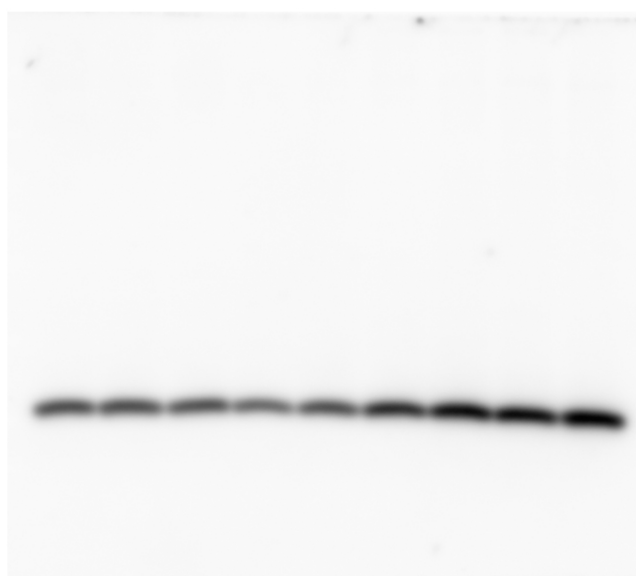

Supplement: Supplementary file 7 — Source Data for Figure 5 [file EMMM-10-e8184-s005.zip › Source_Data_F5/Fig5C_TT.pdf]

A $\beta$

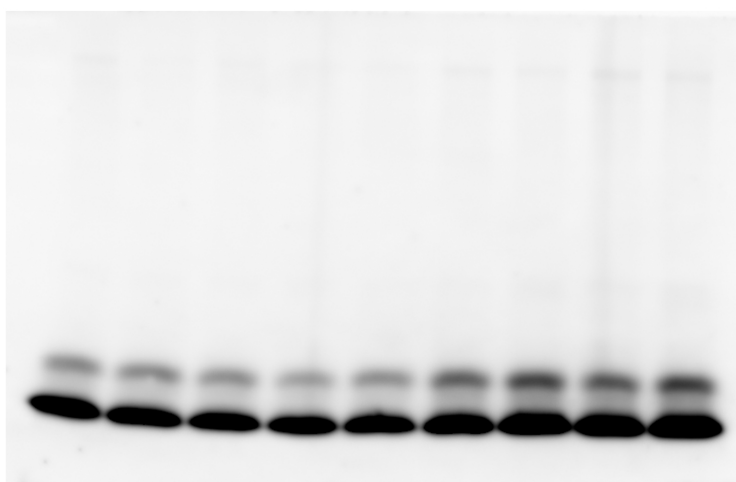

Supplement: Supplementary file 7 — Source Data for Figure 5 [file EMMM-10-e8184-s005.zip › Source_Data_F5/Fig5C_Urea.pdf]
